# Biodiversity and five novel species of myxozoan parasites in *Barbonymus* spp. (Cyprinidae, Cypriniformes) from Malaysia

**DOI:** 10.1038/s41598-025-14254-y

**Published:** 2025-08-09

**Authors:** Nadhirah Syafiqah Suhaimi, Csaba Székely, Kálmán Molnár, Gábor Cech, Boglárka Sellyei, Muhammad Hafiz Borkhanuddin

**Affiliations:** 1 HUN-REN Veterinary Medical Research Institute, Budapest, Hungary; 2 Doctoral School of Animal Biotechnology and Animal Science, Hungarian University of Agriculture and Life Sciences, Gödöllő, Hungary; 3 Faculty of Science and Marine Environment, Universiti Malaysia Terengganu, 21030 Kuala Nerus, Malaysia

**Keywords:** Myxozoa, Diversity, Morphology, 18S rDNA gene, *Barbonymus* spp., Malaysia, Molecular biology, Zoology

## Abstract

Up to this time, only five myxosporean species have been documented from fishes of the *Barbonymus* genus. Due to a limited number of myxozoan studies conducted in Southeast Asia, particularly in Malaysia, the diversity of this parasite group remains largely undiscovered. In this study, a comprehensive parasitology survey was conducted, revealing nine myxozoan parasites, including five different *Myxobolus* spp., three *Thelohanellus* sp. and one *Myxidium* sp. Using morphological and molecular data, we describe here five new species: *Myxobolus gonionoti* n. sp., found in the gill filaments; *Myxobolus barbonymi* n. sp., and *Myxobolus faizahae* n. sp. found in the muscle cells; *Thelohanellus gonionoti* n. sp. found in the fins; and *Thelohanellus barbonymi* n. sp. found in the gill arches. Additionally, we identified spores of the previously described *Myxobolus dykovae* in the gill lamellae of *B. schwanefeldii* and *Thelohanellus zahrahae* in the gill filaments of *B. gonionotus*. Furthermore, two undescribed species were documented solely based on morphology and morphometrics, a *Myxobolus* sp. from the muscle cells of *B. schwanefeldii* and a *Myxidium* sp. from the gallbladder of *B. gonionotus*.

## Introduction

The subphylum Myxozoa^1^ constitutes a diverse group of metazoan parasites characterized by a highly simplified morphology and a complex parasitic life cycle. These parasites play a significant role as pathogens in the wild and cultured fish stocks^2^. Among myxosporeans, the genus *Myxobolus* Bütschli, 1882 is the most diverse, with over 900 known species worldwide^3^. The genus *Myxidium* is the second most diverse, comprising more than 230 species^4^. Meanwhile, the genus *Thelohanellus*, ranking sixth in diversity, has more than 100 nominal species described to date^5^. Despite more than 3000 species having been described globally^6^, the biodiversity of this parasites is significantly understudied especially in Southeast Asia and Africa, where just a few studies on myxozoans have been conducted. This is largely due to limited numbers of experts specializing in myxozoan parasites. Data on myxosporeans, from countries in Southeast Asia such as Brunei, Cambodia, Laos, East Timor, Singapore, and the Philippines are still completely lacking. However, interest in myxozoan research has been steadily increasing worldwide, as evidenced by the growing amount of publications describing new species^3,7–12^.

The genus *Barbonymus* comprises ten species, including *Barbonymus schwanefeldii* Bleeker, 1854, *Barbonymus gonionotus* Bleeker, 1849, *Barbonymus altus* Günther, 1868, *Barbonymus belinka* Bleeker, 1860, *Barbonymus balleroides* Valenciennes, 1842, *Barbonymus collingwoodii* Günther, 1868, *Barbonymus mahakkamensis* Ahl, 1922, *Barbonymus platysoma* Bleeker, 1855, *Barbonymus strigatus* Boulenger, 1894, and *Barbonymus sunieri* Weber et Beaufort, 1916^13–16^. These species are widely distributed across Southeast Asia^17–21^. In Malaysia, three species—*B. schwanefeldii*, *B. gonionotus* and *B. altus* are particularly common in freshwater ecosystems. These species are not only ecologically important but also hold significant commercial value, both as ornamental fish and as food, due to their high market price and consumer preference^22,23^. Given the economic importance of these species, conducting parasite surveys is crucial to mitigate the risk of pathogen infections that could affect both wild and cultured fish populations.

To our knowledge, only five myxozoan species have been reported from *Barbonymus* spp. in Malaysia so far. These include *Thelohanellus zahrahae* Székely, Shaharom-Harrison, Cech, Mohamed et Molnár 2009^24^; *Thelohanellus catlae* Chakravarty et Basu, 1948; and *Myxobolus macrocapsularis* Reuss 1906, which infect the gills of Java barb, *B. gonionotus*^25^. Additionally, *Myxobolus dykovae* Székely, Shaharom-Harrison, Cech, Ostoros et Molnár 2009, has been documented infecting the gill lamellae of tinfoil barb, *B. schwanefeldii*^26^, while the recently described *Ceratomyxa schwanefeldii* Suhaimi, Székely, Cech, Sellyei et Borkhanuddin 2025, was found infecting the gallbladder of the same fish^27^. However, no myxozoan species have been already reported from red tailed tinfoil barb, *B. altus*. Considering the limited number of myxozoan species detected from Java barb, tinfoil barb and red-tailed tinfoil barb in Malaysia, a comprehensive survey was conducted on these species. In this study, we describe five novel myxosporean species: *Myxobolus gonionoti* n. sp., *Myxobolus barbonymi* n. sp., *Myxobolus faizahae* n. sp., *Thelohanellus gonionoti* n. sp., and *Thelohanellus barbonymi* n. sp. using morphological and molecular analyses. Additionally, we report on the occurrence of two previously known species, *T. zahrahae* and *Myxobolus dykovae,* in Malaysia and two unidentified myxozoan species, a *Myxobolus* sp. and a *Myxidium* sp.

## Materials and methods

### Ethical statement

The fish handling, sampling, and dissecting were approved by the Institutional Animal Care and Use Committee of the Veterinary Medical Research Institute, Budapest, Hungary. All research involving experiments on fish from genus *Barbonymus* was reviewed and approved by the Hungarian National Scientific Ethical Committee on Animal Experimentation under reference number: PE/EA/00081-4/2023. The authors complied with the ARRIVE guidelines (https://arriveguidelines.org).

### Fish sampling and microscopic study

A total of twenty-eight fish samples (with a total length of 10.0–30.0 cm) from the rivers of Sungai Tong, Setiu and Sungai Nerus, Kuala Nerus were purchased from a local fish market in Kuala Terengganu, Terengganu, Malaysia (5° 18′ 44.703″ N 103° 7′ 40.332″ E). The procurement was conducted at two-week intervals over two periods: July to August 2023 and September to November 2024. The live fish were transported to the Marine Science Biodiversity Laboratory of the Faculty of Science and Marine Environment at Universiti Malaysia Terengganu in oxygenated plastic bags. Upon arrival, they were kept in a tank with continuous aeration until autopsy. Taxonomic identification of the fish specimens was based on their morphology. In the laboratory, the fish were euthanized by neural pithing, dissected, and all organs were thoroughly examined to detect plasmodia of myxosporean parasites. These studies were initially conducted with the naked eye, followed by observations under a dissecting microscope and a light microscope to confirm the presence of the plasmodia.

The plasmodia containing spores were isolated from infected organs and tissues, and examined on slide under an Olympus CX33 biological microscope (Olympus Corporation, Japan). Following microscopic observation, the remaining plasmodia and spores were collected into 1.5 mL Eppendorf tubes and preserved in 90% ethanol for molecular studies, and in 10% neutral buffered formalin for subsequent morphological analysis. All fixed samples were later transported to the Fish Pathology and Parasitology Laboratory at the HUN-REN Veterinary Medical Research Institute (Budapest, Hungary), where further analyses were conducted, including high-magnification examinations (100 × objective) and photographic documentation with an Olympus BX53 light microscope equipped with an Olympus DP74 digital camera (Olympus Corporation, Japan). The spores were measured according to the guidelines of Lom and Dyková^28^, using ImageJ software (http://imagej.nih.gov/ij). All measurements are presented as the mean and standard deviation (SD), followed by the range in parentheses in micrometers (µm). For histological analysis, infected organ and tissues fixed in 10% neutral buffered formalin (NBF) were embedded in paraffin, sectioned at 3–4 µm thin, stained with hematoxylin and eosin (H&E), and subsequently examined and photographed using an Olympus DP74 digital camera.

### Molecular and phylogenetic analyses

DNA was extracted from plasmodia and spores that had been preserved in 90% ethanol. Prior to extraction, the samples were washed and centrifuged following the method described by Suhaimi et al.^29,30^. Genomic DNA was then isolated using the Geneaid Tissue Genomic DNA Mini Kit (Geneaid Biotech Ltd., Taiwan), according to the manufacturer’s instructions for animal tissue samples. The 18S rDNA gene was subsequently amplified via direct PCR using various primer sets listed in Table 1. All polymerase chain reactions (PCRs) were performed in 25 μL reaction volumes, containing 2 μL of template DNA, 1 × DreamTaq buffer (10 × ; Thermo Scientific), 0.2 mM dNTP mix (10 mM; Thermo Scientific), 1.25 U DreamTaq polymerase (5 U; Thermo Scientific), 12.5 pmol of each primer, and molecular grade water. PCR amplifications using different primer pairs were conducted under various thermal cycling conditions. For the MyxospecF-18R and 18E-MyxospecR primers, the protocol of Liu et al.^31^ was followed, with a modified elongation time of 90 s. Amplifications using ERIB1-ACT1R primers followed the method described by Úngari et al.^32^. PCR products were analyzed by agarose gel electrophoresis. Bands of the expected size were excised, purified using the DNA Fragment Purification Kit (InViTek GmbH, Berlin, Germany), and sequenced in both directions using the BigDye Terminator v3.1 Cycle Sequencing Kit (Applied Biosystems, Foster City, CA, USA). Sequencing was performed on an ABI PRISM 3100 Genetic Analyzer, using the primers listed in Table 1.

**Table 1:** Primers used for PCR amplification or sequencing of 18S rDNA of each myxozoan species.

Species	Primer	Application	References
*Myxobolus gonionoti* n. sp.	ERIB1	PCR and sequencing	^33^
	ACT1R	PCR and sequencing	^34^
	18R	PCR and sequencing	^35^
	MyxospecF	PCR and sequencing	^36^
	Myxgen4R	Sequencing	^37^
*Myxobolus barbonymi* n. sp.	ERIB1	PCR and sequencing	^33^
	ACT1R	PCR and sequencing	^34^
	18R	PCR and sequencing	^35^
	MyxospecF	PCR and sequencing	^36^
	Myxgen4F	Sequencing	^38^
	Myxgen4R	Sequencing	^37^
*Myxobolus faizahae* n. sp.	ERIB1	PCR and sequencing	^33^
	ACT1R	PCR and sequencing	^34^
	18R	PCR and sequencing	^35^
	MyxospecF	PCR	^36^
	ACT1F	Sequencing	^34^
	Myxgen4R	Sequencing	^37^
*Thelohanellus gonionoti* n. sp.	ERIB10	PCR and sequencing	^33^
	Myx1F	PCR and sequencing	^34^
	ACT1fr	Sequencing	^34^
	ACT1R	Sequencing	^34^
	Myxgen4F	Sequencing	^38^
*Thelohanellus barbonymi* n. sp.	ERIB1	PCR and sequencing	^33^
	ACT1R	PCR and sequencing	^34^
	18R	PCR and sequencing	^35^
	MyxospecF	PCR and sequencing	^36^
	Myxgen4F	Sequencing	^38^
	Myxgen4R	Sequencing	^37^
*Thelohaellus zahrahae*	ERIB1	PCR and sequencing	^33^
	ACT1R	PCR and sequencing	^34^
	18R	PCR and sequencing	^35^
	MyxospecF	PCR and sequencing	^36^
	Myxgen4F	Sequencing	^37^
*Myxobolus dykovae*	18R	PCR and sequencing	^35^
	MyxospecF	PCR and sequencing	^36^
	18E	PCR and sequencing	^39^
	ACT1R	Sequencing	^34^
	Myxgen4F	Sequencing	^38^
	Myxgen4R	Sequencing	^37^

The 18S rDNA sequences were analyzed using Geneious Prime v11.1 (Bio-matters; Auckland, New Zealand; https://www.geneious.com/)^40^ for visualization, editing, and assembly. Phylogenetic analysis included seven sequences generated in this study along with 33 related sequences obtained from the GenBank database, using *Chloromyxum cyprini* (AY604198) as the outgroup. Multiple sequence alignment was performed using the ClustalW algorithm^41^ in MEGA X (Molecular Evolutionary Genetics Analysis, 64-bit version; https://www.megasoftware.net/)^42^, and phylogenetic trees were constructed using both the Maximum Likelihood method with 1000 bootstrap replicates in MEGA X, and Bayesian Inference in MrBayes v3.2.4^43^. Gaps were treated using partial deletion with 75% site coverage cut-off. The analyses were performed using the GTR + G + I model based on the Akaike information criterion (AIC) and Bayesian Information Criterion (BIC) executed in jModelTest 2.1.10 v20160303^44^. In the Bayesian analysis, Markov Chain Monte Carlo (MCMC) chains were executed for 1,000,000 generations, with the initial 25% of the generations were discarded to produce a consensus tree from the remaining topologies. Bootstrap values (BS) ≥ 70% for ML and posterior probabilities (PP) ≥ 0.90 for BI analyses were considered as well-supported clade. The resulting topologies were visualized in MEGA X and FigTree v. 1.4.4^45^ and graphically edited with Inkscape (Free Software Foundation, Inc., MA, USA). The pairwise distances were performed using a distance matrix model *p*-distance for transitions and transversions in MEGA X software package.

## Results

For the survey of myxosporean parasites in Malaysia, three *Barbonymus* species were selected. Among the six *B. gonionotus* examined, all were found to be infected (100%). In contrast, only one out of six *B. schwanefeldii* individuals was infected (16.6%). For *B. altus*, seven out of sixteen individuals found positive for myxosporean infections, resulting in a prevalence of 43.7%. Plasmodia were found in various body parts, including the gill filaments, gill lamellae, gill arches, muscle, fins, and gallbladder. Only mature spores were observed within the examined plasmodia. In total, five new myxozoans species were found, comprising three *Myxobolus* spp.—*M. gonionoti* n. sp., *M. barbonymi* n. sp., and *M. faizahae* n. sp. and two *Thelohanellus* species—*T. gonionoti* n. sp. and *T. barbonymi* n. sp. Additionally, two previously known species; *T. zahrahae* and *M. dykovae* were identified, along with an unidentified *Myxobolus* sp. and a *Myxidium* sp. In terms of myxosporeans prevalence, the novel *T. gonionoti* n. sp. showed the highest (50.0%, 3/6) and *T. barbonymi* n. sp. showed the lowest (12.5%, 2/16) prevalence. Furthermore, none of the infected fish collected from Sungai Tong, Setiu and Sungai Nerus, Kuala Nerus, Terengganu, Malaysia displayed any clinical symptoms.

### *Myxobolus gonionoti* n. sp.

**Fig. 1:**
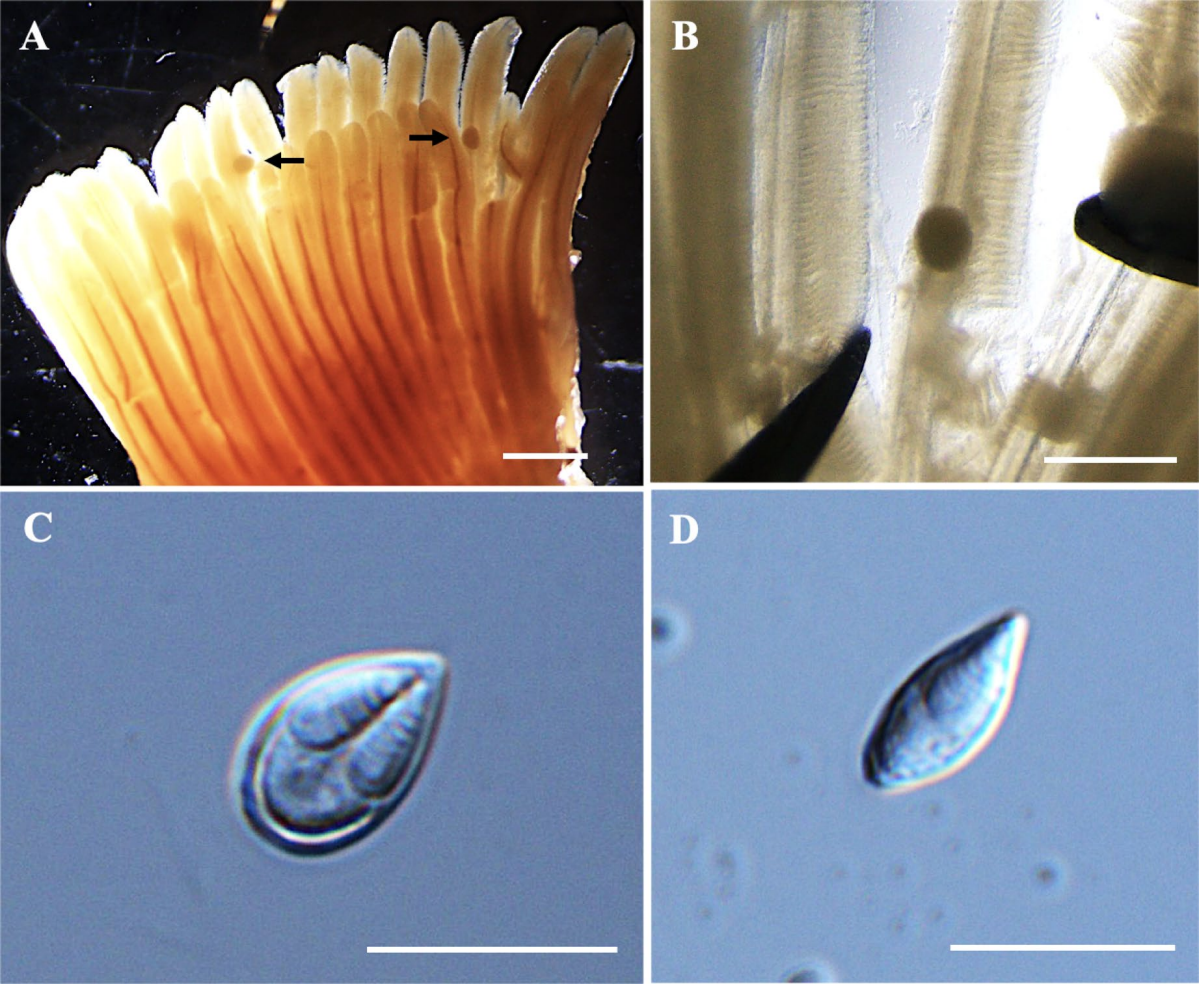
Photomicrographs of *Myxobolus gonionoti* n. sp. from the gills of *Barbonymus gonionotus*. (**A**) Plasmodia (black arrow) of *M. gonionoti* n. sp. in the formalin-fixed gill filaments. (**B**) Round plasmodium located at the central part of a filament. (**C**) Spore in frontal view. (**D**) Spore in sutural view. Scale bars represent 10 µm, except (**A**) 1 mm and (**B**) 500 µm.

*Plasmodia*: Found on the gill filament, histozoic, big and round in shape (Fig. 1A–B), measuring 0.3 mm in length and 0.2 mm in width (*n* = 2).

*Description of myxospores*. Fixed spores oval in both frontal and sutural views, tapering at the anterior ends (Figs. 1C–D, 9A) measuring 9.3 ± 0.3 (8.2–9.8) µm in length, 6.1 ± 0.3 (5.4–6.6) µm in width, and 4.7 ± 0.3 (4.4–5.1) µm in thickness (*n* = 9). Two pyriform, closely equal polar capsule sizes occupying half of the spore body cavity. Larger polar capsule measuring 5.2 ± 0.3 (4.3–6.0) µm in length and 2.1 ± 0.2 (1.6–2.4) µm in width and smaller polar capsule measuring 4.9 ± 0.3 (3.9–5.4) µm in length and 1.9 ± 0.2 (1.6–2.3) µm in width. No intercapsular appendix observed. Seven polar tubule coils in larger polar capsules and six in smaller polar capsule, positioned perpendicularly to the longitudinal axis of the polar capsules. Sporoplasm binucleate and containing an iodinophilous vacuole, but no observable mucous envelope. Measurements from 30 formalin-fixed spores from one host.

### Taxonomic summary

Type host: Java barb, *Barbonymus gonionotus*.

Locality: Sungai Tong, Setiu, Terengganu, Malaysia.

Site of infection: Gill filament.

Prevalence: 16.6% (1/6).

Type material: Phototypes were deposited in the parasitological collection of the Zoological Department, Hungarian Natural History Museum, Budapest, Coll. No. HNMPCC-HNHM-PAR-72091.

Etymology: The name *Myxobolus gonionoti* n. sp. was derived from the name of the fish host, *Barbonymus gonionotus*.

18S rDNA sequence: Partial 18S rDNA sequence of *M. gonionoti* n. sp., consisting of 1,917 base pairs was deposited in GenBank under the accession number PV665937. The 18S rDNA sequence from the *M. gonionoti* n. sp. did not significantly match any of the myxozoan sequences available in GenBank. Pairwise distance estimation of the newly obtained 18S rDNA sequence indicated the highest similarities by 91.2% to *M. barbonymi* n. sp. (PV665939) (Table 6). Phylogenetic analysis revealed that *M. gonionoti* n. sp. clustered with maximum bootstrap support within a clade of muscle-infecting *Myxobolus* spp. that primarily infect cyprinids. However, its position is basal and separated from the rest of the clade (Fig. 10).

Remarks: The closest morphological resemblance to *M. gonionoti* n. sp. was found with *M. dykovae*^26^, although notable morphometric differences were present (Table 2). Myxospore of *M. gonionoti* n. sp. was smaller than that of *M. dykovae* but *M. gonionoti* n. sp. had unequal polar capsule sizes, whereas *M. dykovae* had equal polar capsule sizes. In terms of morphometric measurements, *M. gonionoti* n. sp. showed the greatest similarities to *Myxobolus sangei* Fomena, Lekeufack Folefack et Tang, 2007 particularly in spore width, large polar capsule width, and small polar capsule length and width. However, *M. gonionoti* n. sp. had smaller spore and polar capsule lengths. The number of turns in the larger polar capsule of *M. gonionoti* was similar to that of *M. sangei*, but it differed in the smaller polar capsule. Additionally, the spore width and larger polar capsule width of *M. gonionoti* n. sp. were similar to those of *Myxobolus carlhubbsi* McAllister, Cloutman, Leis, Camus et Robinson, 2023. Notably, Ky and Te^25^ in Chinh et al.^46^ reported *Myxobolus macrocapsularis* Reuss, 1849 from the gills of *B. gonionotus*, is a common parasite of the common bream (*Abramis brama*) and white bream (*Blicca bjoerkna*) in Europe. When comparing our spores with *M. macrocapsularis* as described by Ky and Te^25^, significant differences were observed particularly in the polar capsules; present spores have slightly unequal-sized polar capsules, while *M. macrocapularis* has equal-sized polar capsules. Additionally, the spores of *M. macrocapularis* differ from those of *M. gonionoti* n. sp. in the shape of their anterior ends; *M. macrocapularis* has slightly convergent or papilla-like anterior ends, whereas *M. gonionoti* n. sp. has tapered anterior ends. However, *M. gonionoti* n. sp. was compared with all available descriptions of *M. macrocapularis* in the literature^47^. The spores exhibited morphological and morphometric similarities to those of *M. macrocapularis* described by Donec and Shulman^48^. Notable, the schematic drawing by Donec and Shulman^48^ indicates that *M. macrocapsularis* has unequal-sized polar capsules, yet the descriptions only provide measurements for one size of polar capsule, suggesting similar sizes.

**Table 2:** Comparative data for myxospore measurements (mean value and standard deviation (SD) followed by the range in parentheses) of *Myxobolus gonionoti n.* sp., *Myxobolus dykovae* and species with tapered anterior ends. All measurements are in μm. SL spore length, SW spore width, ST spore thickness, PCL polar capsule length, PCW polar capsule width, PFC polar filament coils.

Species	Host	Site infection	SL	SW	ST	Large/Equal		Small		PCW	PCF	References
						PCL	PCW	PFC	PCL			
***Myxobolus gonionoti* n. sp.**	***Barbonymus gonionotus***	**Gill lamellae**	9.3 ± 0.3 (8.2–9.8)	6.1 ± 0.3 (5.4–6.6)	4.7 ± 0.3 (4.4–5.1)	5.2 ± 0.3 (4.3–6.0)	2.1 ± 0.2 (1.6–2.4)	7	4.9 ± 0.3 (3.9–5.4)	1.9 ± 0.2 (1.6–2.3)	6	**Present study**
*M. sangei*	*Brycinus macrolepidotus*	Gill, skin, kidney	10.1 (9.0–10.5)	6.2 (6.0–6.8)	–	6.2 (5.7–7.0)	2.2 (2.0–3.0)	7–8	4.8 (4.0–5.5)	1.7 (1.5–2.0)	4–5	^49^
*M. macrocapsularis*	*Barbonymus gonionotus*	Gills	(10.9–11.6)	7.2	6.2	6.2	2.3	–	–	–	–	^25^
*M. dajiangensis*	*Cyprinus carpio*	Gill lamellae	14.8 ± 0.4 (13.9–15.6)	8.0 ± 0.5 (7.3–9.1)	5.5	8.0 ± 0.4 (7.1–8.8)	2.5 ± 0.2 (2.0–3.2)	9–11	7.4 ± 0.4 (6.1–8.0)	2.5 ± 0.2 (2.0–3.2)	9–11	^50^
*M. carlhubbsi*	*Luxilus chrysocephalus isolepis*	Gill filaments	12.7 ± 0.4 (12.0–13.4)	6.1 ± 0.3 (5.0–6.6)	5.4 ± 0.3 (5.1–6.0)	6.4 ± 0.3 (5.7–7.3)	2.1 ± 0.2 (1.8–2.3)	6–11	6.2 ± 0.3 (5.2–6.8)	2.2 ± 0.1 (1.8–2.5)	6–11	^51^
*M. voremkhai*	*Tachysurus fulvidraco*	Gill arches	14.3 ± 0.6 (10.4–12.6)	7.7 ± 0.4 (6.9–8.4)	5.7 ± 0.6 (4.9–6.8)	6.2 ± 0.4 (5.4–7.1)	2.7 ± 0.2 (2.5–3.0)	5–7	5.9 ± 0.3 (5.3–6.5)	2.5 ± 0.2 (2.2–2.8)	5–7	^52^
***M. dykovae***	***Barbonymus schwanefeldii***	**Gill lamellae**	11.7 ± 0.6 (10.3–12.8)	6.8 ± 0.4 (5.9–7.7)	5.4 ± 0.3 (5.1–5.7)	5.8 ± 0.5 (4.7–7.0)	2.2 ± 0.2 (1.8–2.6)	6–7	–	–	–	**Present study**
*M. dykovae*	*Barbonymus schwanefeldii*	Gill lamellae	12.0 ± 0.5 (11.0–12.7)	6.2 ± 0.4 (5.6–6.7)	5.8 ± 0.16 (5.7–6.0)	6.0 ± 0.07 (5.9–6.1)	2.1 ± 0.14 (2.0–2.3)	6–7	–	–	**–**	^26^
*M. alvarezae*	*Leuciscus idus*	Gill filaments	11.7 ± 0.42 (11.3–12.6)	6.8 ± 0.31 (6.5–7.6)	6.4 ± 0.41 (6.1–6.9)	6.7 ± 0.32 (6.1–7.2)	2.3 ± 0.16 (2.0–2.5)	6	–	–	–	^53^
*M. koi*	*Carassius auratus*	Gills	13.6 ± 0.6 (12.3–15.1)	8.3 ± 0.4 (7.6–9.1)	–	7.4 ± 0.5 (6.4–8.5)	2.8 ± 0.2 (2.4–3.4)	9–10	–	–	–	^54^

### *Myxobolus barbonymi* n. sp.

**Fig. 2:**
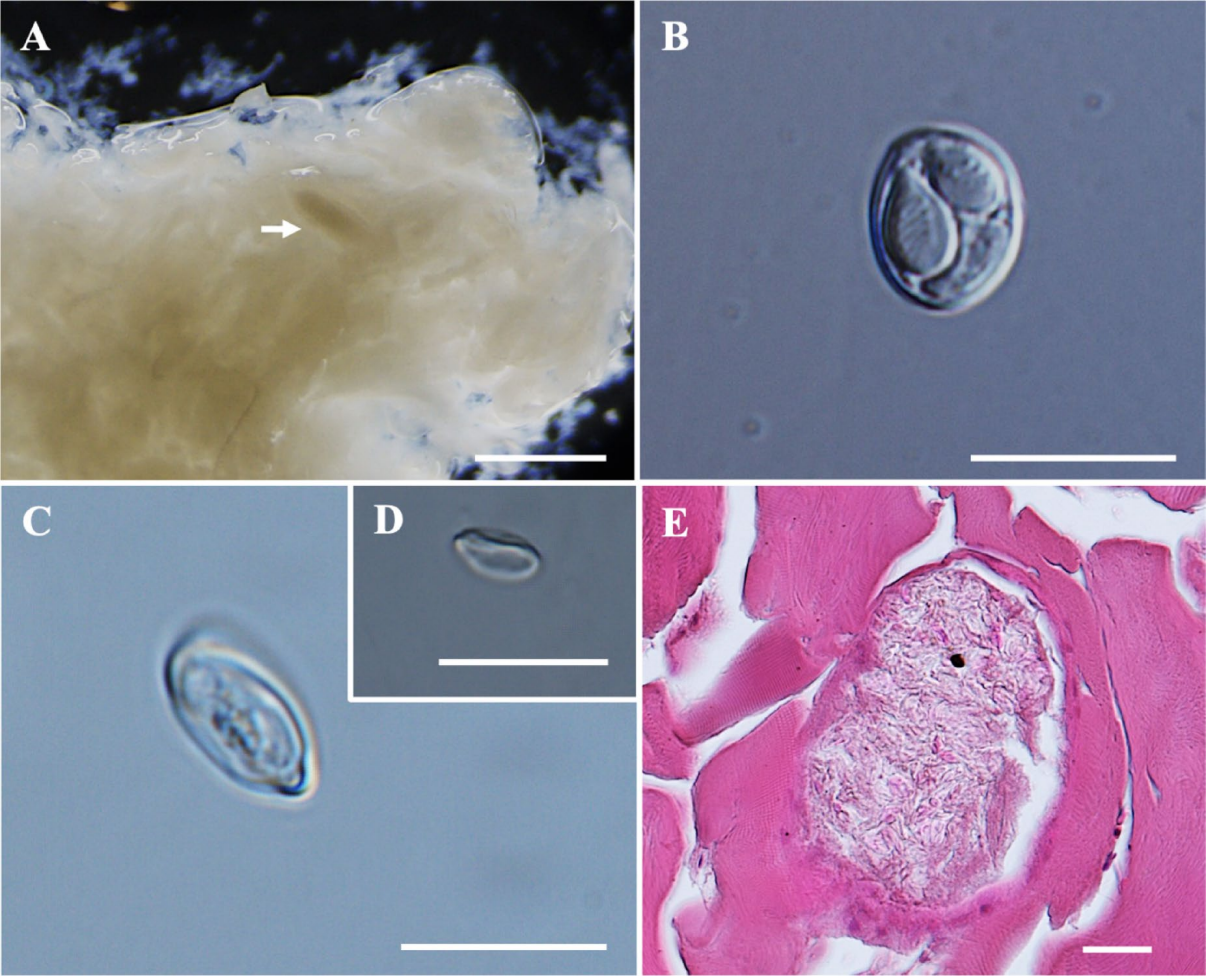
Photomicrographs of *Myxobolus barbonymi* n. sp. from the skeletal muscle of *Barbonymus gonionotus*. (**A**) Plasmodia (white arrow) of *M. barbonymi* n. sp. in formalin-fixed muscle. (**B**) Spore in frontal view. (**C**) Spore in sutural view. (**D**) Sutural line of the spore. (**E**) Histological transverse section of muscle cells showing an intracellular plasmodium (P) of *M. barbonymi* n. sp. within a (skeletal) muscle cell of *B. gonionotus*, stained with hematoxylin and eosin (H&E). Scale bars represent 10 µm, except (**A**) 1 mm, (**D**) 20 µm and (**E**) 20 µm.

*Plasmodia*: Found in the muscle, histozoic, elongated in shape (Fig. 2A), measuring 0.8 mm in length and 0.2 mm in width (*n* = 2).

*Description of myxospores:* Fixed spores ellipsoidal, asymmetrical in frontal view, with flattened anterior end and lemon-shaped in sutural view (Figs. 2B–D, 9E) measuring 8.4 ± 0.5 (7.4–9.9) µm in length, 6.9 ± 0.5 (6.1–7.8) µm in width, and 5.2 ± 0.2 (4.9–5.4) µm in thickness (*n* = 5). Two pyriform and unequal polar capsules; larger polar capsule measuring 4.9 ± 0.3 (4.2–5.5) µm in length and 2.9 ± 0.3 (2.4–3.4) µm in width and smaller polar capsule measuring 3.8 ± 0.4 (3.0–4.7) µm in length and 2.4 ± 0.2 (2.0–2.8) µm in width. Strong and ‘U’-shaped intercapsular appendix between the anterior ends of the polar capsules. Polar tubule coiling five to six times in larger polar capsules and three to four times in smaller polar capsule, positioned perpendicularly to the longitudinal axis of the polar capsules. Sporoplasm binucleate, no iodinophilous vacuole and mucous envelope. Measurements from 30 formalin-fixed spores from two hosts.

### Taxonomic summary

Type host: Java barb, *Barbonymus gonionotus*.

Locality: Sungai Tong, Setiu, Terengganu, Malaysia.

Site of infection: Intracelullarly in the muscle cell.

Prevalence: 33.3% (2/6).

Type material: Phototypes were deposited in the parasitological collection of the Zoological Department, Hungarian Natural History Museum, Budapest, Coll. No. HNMPCC-HNHM-PAR-72089.

Etymology: The name *Myxobolus barbonymi* n. sp. was derived from the genus name of the fish host, *Barbonymus gonionotus*.

Histology: Histopathological analysis revealed that the plasmodium of *M. barbonymi* n. sp. developed intracellularly within muscle cells (Fig. 2E). The lack of young sporogonic stages confirmed that the plasmodium was mature and completely filled with mature spores.

18S rDNA sequence: Partial 18S rDNA sequence of *M. barbonymi* n. sp., consisting of 1,849 base pairs was deposited in GenBank under the accession number PV665939. The 18S rDNA sequence from the *M. barbonymi* n. sp. did not significantly match any of the myxozoan sequences available in GenBank. Pairwise distance estimation of the newly obtained 18S rDNA sequence indicated the highest similarities by 92.7% to *Myxobolus pseudodispar* (KU340979) and 92.6% to *Myxobolus musculi* (JQ388891) (Table 6). Phylogenetic analysis revealed that *M. barbonymi* n. sp. was positioned in a monophyletic clade basally to other muscle-infecting *Myxobolus* spp. with high bootstrap support (Fig. 10).

Remarks: The morphology and morphometrics of *M. barbonymi* n. sp. differed from any previously described *Myxobolus* spp. with unequal polar capsule sizes. The closest morphological resemblance to *M. barbonymi* n. sp. was found with *Myxobolus tauricus* Miroshnichenko, 1979 and *Myxobolus hakyi* Landsberg et Lom, 1991. Furthermore, *M. barbonymi* n. sp. can be distinguished by its flattened anterior end, a feature not previously reported in any *Myxobolus* spp. nor any muscle-infecting *Myxobolus* species. The morphometric measurements of *M. barbonymi* n. sp. were almost similar to *Myxobolus faizahae* n. sp., with *M. barbonymi* n. sp. being larger in size. Notably, the wider polar capsule and shorter polar capsule of *M. barbonymi* n. sp. were similar to those of *M. pseudodispar*^55,56^ (Table 3).

**Table 3:** Comparative data for myxospore measurements (mean value and standard deviation (SD) followed by the range in parentheses) of *Myxobolus barbonymi* n. sp., *Myxobolus faizahae* n. sp., *Myxobolus* sp. and species with unequal polar capsules. All measurements are in μm. SL spore length, SW spore width, ST spore thickness, PCL polar capsule length, PCW polar capsule width, PFC polar filament coils.

Species	Host	Site infection	SL	SW	ST	Large		Small		PCW	PCF	References
						PCL	PCW	PFC	PCL			
***Myxobolus barbonymi* n. sp.**	***Barbonymus gonionotus***	**Muscle**	8.4 ± 0.5 (7.4–9.9)	6.9 ± 0.5 (6.1–7.8)	5.2 ± 0.2 (4.9–5.4)	4.9 ± 0.3 (4.2–5.5)	2.9 ± 0.3 (2.4–3.4)	5–6	3.8 ± 0.4 (3.0–4.7)	2.4 ± 0.2 (2.0–2.8)	3–4	**Present study**
***Myxobolus faizahae* n. sp.**	***Barbonymus altus***	**Muscle**	9.3 ± 0.3 (8.7–9.9)	5.8 ± 0.2 (5.4–6.1)	4.4 ± 0.3 (3.5–4.8)	4.4 ± 0.3 (3.8–5.0)	2.8 ± 0.3 (2.3–3.3)	4–5	3.3 ± 0.2 (2.9–3.9)	1.9 ± 0.1 (1.6–2.2)	3–4	**Present study**
***Myxobolus* sp.**	***Barbonymus schwanefeldii***	**Muscle**	11.7 ± 0.5 (11.0–12.5)	6.4 ± 0.5 (5.8–7.2)	4.8 ± 0.8 (4.3–5.4)	6.0 ± 0.5 (5.3–6.9)	2.8 ± 0.3 (2.2–3.2)	5	4.8 ± 0.4 (4.2–5.4)	2.2 ± 0.3 (1.7–2.9)	4	**Present study**
*M. bhadrensis*	*Catla catla*	Muscle	10.0 ± 0.4 (9.2–10.4)	6.6 ± 0.3 (6.0–7.2)	4.5 ± 0.5 (4.0–5.3)	5.5 ± 0.3 (4.8–6.0)	2.0 ± 0.2 (1.6–2.6)	4	4.2 ± 0.4 (3.6–4.8)	2.0 ± 0.2 (1.6–2.6)	3	^57^
*M. pseudodispar*	*Rutilus rutilus, Blicca joerkna, Abramis brama*	Muscle	12.2 ± 0.8 (11.0–13.6)	7.0 ± 0.7 (5.8–8.3)	5.6 ± 0.3 (5.0–6.0)	5.9 ± 0.3 (5.6–6.6)	2.9 ± 0.3 (2.1–3.3)	4–6	3.8 ± 0.4 (3.5–5.0)	2.8 ± 0.1 (2.7–3.0)	3–4	^55,56^
*M. cyprini*	*Cyprinus carpio*	Muscle	(10.0–16.0)	(8.0–12.0)	–	(5.2–7.0)	–	–	–	–	–	^58^
*M. musculi*	*Barbus barbus*	Muscle	11.7 ± 0.6 (11.0–13.0)	9.4 ± 0.4 (8.7–9.8)	5.6 ± 0.4 (4.8–5.6)	6.7 ± 0.4 (6.2–7.3)	3.4 ± 0.4 (2.9–3.8)	4–5	5.9 ± 0.6 (5.3–6.6)	2.9 ± 0.5 (2.1–3.3)	3	^59^

### *Myxobolus faizahae* n. sp.

**Fig. 3:**
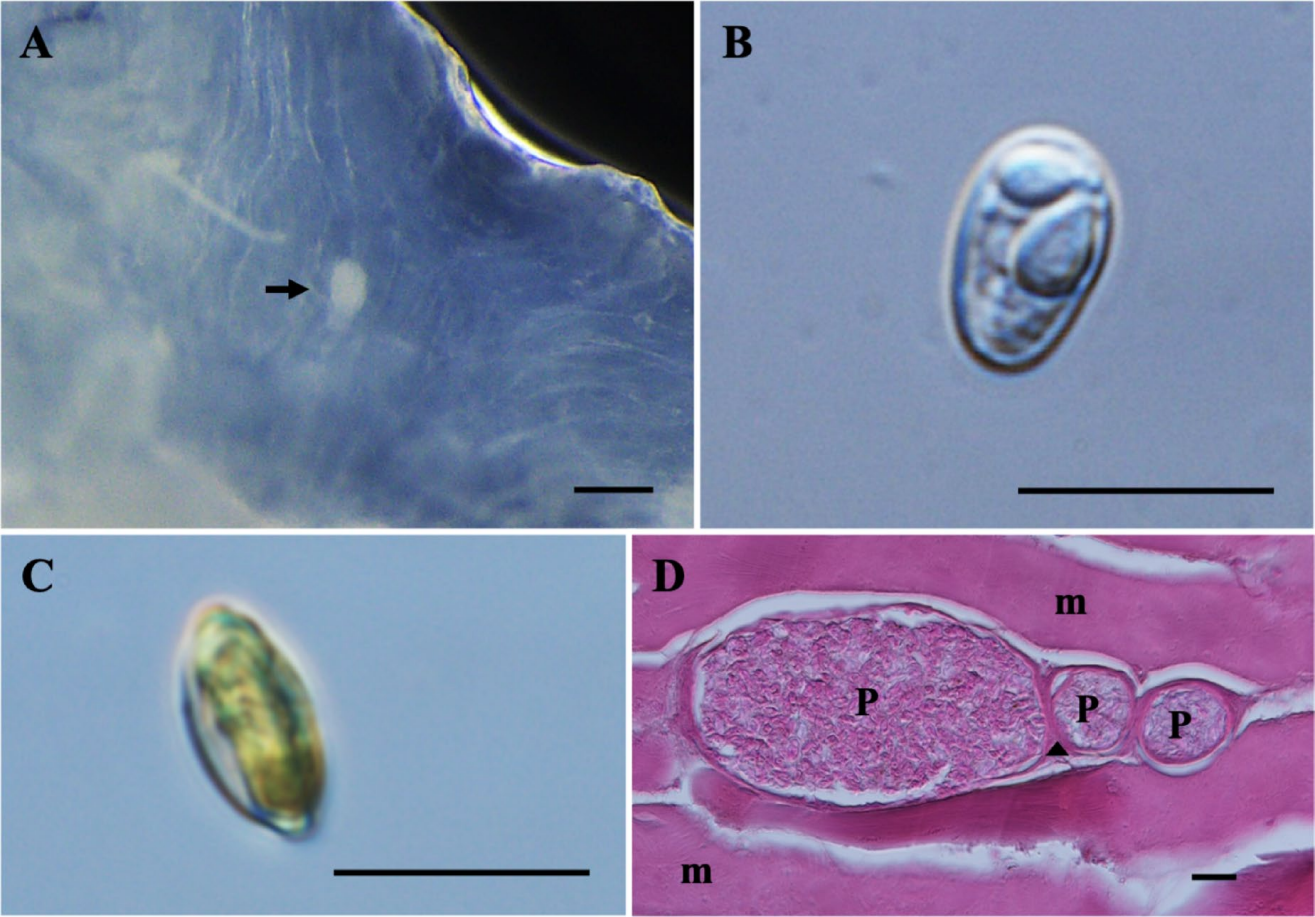
Photomicrographs of *Myxobolus faizahae* n. sp. from the muscle of *Barbonymus altus*. (**A**) Plasmodia (black arrow) of *M. faizahae* n. sp. in formalin-fixed muscle. (**B**) Spore in frontal view. (**C**) Spore in sutural view. (**D**) Histological longitudinal section of muscle cells showing plasmodia (P) located among (skeletal) muscle cells (m) of *B. altus*, surrounding connective tissue (arrowhead), stained with hematoxylin and eosin (H&E). Scale bars represent 10 µm, except (**A**) 200 µm and (**D**) 20 µm.

*Plasmodia*: Found between muscle cells, histozoic, elongated and oval in shape (Fig. 3A), measuring 92.9 ± 62.7 µm in length and 59.3 ± 29.9 µm in width (*n* = 4).

*Description of myxospores*: Fixed spores ellipsoidal, asymmetrical in frontal view and lemon-shaped in sutural view (Figs. 3B–C, 9B) measuring 9.3 ± 0.3 (8.7–9.9) µm in length, 5.8 ± 0.2 (5.4–6.1) µm in width, and 4.4 ± 0.3 (3.5–4.8) µm in thickness. Two pyriform and unequal polar capsules; larger polar capsule measuring 4.4 ± 0.3 (3.8–5.0) µm in length and 2.8 ± 0.3 (2.3–3.3) µm in width and smaller polar capsule measuring 3.3 ± 0.2 (2.9–3.9) µm in length and 1.9 ± 0.1 (1.6–2.2) µm in width. No intercapsular appendix observed. Polar tubule coiling four to five times in larger polar capsules and three to four times in smaller polar capsule, positioned perpendicularly to the longitudinal axis of the polar capsules. Sporoplasm binucleate, but iodinophilous vacuole not visible, and no mucous envelope observable. Measurements from 30 formalin-fixed spores from two host.

### Taxonomic summary

Type host: Red tailed tinfoil barb, *Barbonymus altus*.

Locality: Sungai Tong, Setiu and Sungai Nerus, Kuala Nerus, Terengganu, Malaysia.

Site of infection: Associated with intramuscular connective tissues.

Prevalence: 66.6% (4/6).

Type material: Phototypes were deposited in the parasitological collection of the Zoological Department, Hungarian Natural History Museum, Budapest, Coll. No. HNMPCC-HNHM-PAR-72090.

Etymology: The name *Myxobolus faizahae* n. sp. is dedicated to Professor Emeritus Dr. Faizah Shaharom, a renowned fish parasitologist from Malaysia.

Histology: Histopathological analysis revealed that the plasmodium was encapsulated by a thin layer of connective tissue and associated with the intramuscular connective tissue among muscle cells (Fig. 3D).

18S rDNA sequence: Partial 18S rDNA sequence of *M. faizahae* n. sp., consisting of 1,783 base pairs was deposited in GenBank under the accession number PV665938. The 18S rDNA sequence from the *M. faizahae* n. sp. did not significantly match any of the myxozoan sequences available in GenBank. Pairwise distance estimation of the newly obtained 18S rDNA sequence indicated the highest similarities of 94.4% to *Myxobolus bhadrensis* (AF378343) and 94.2% to *Myxobolus kingchowensis* (MH521302) (Table 6). Phylogenetic analysis revealed that *M. faizahae* n. sp. formed a close relationship with *Myxobolus terengganuensis* and clustered within well-supported clade of muscle-infecting *Myxobolus* spp. with high bootstrap support (Fig. 10).

Remarks: The closest morphological resemblance to *M. faizahae* n. sp. was found to *M. pseudodispar*^55,56^ although notable morphometric differences were observable (Table 3). Myxospore of *M. faizahae* n. sp. was smaller than *M. pseudodispar*, they were similar only in the width of the larger polar capsule and in the number of polar tubule coils. In terms of morphometric measurements, *M. faizahae* n. sp. showed the greatest similarity to *M. bhadrensis* Székely, Cech, Chaudhary, Borzák Singh et Molnár, 2015 particularly in spore thickness, smaller polar capsule width and the number of coils in both polar capsules. However, overall, *M. faizahae* n. sp. was smaller than *M. bhadrensis*. The *M. barbonymi* n. sp. was between the *Myxobolus* sp. described here and *M. faizahae* n. sp. in size. Additionally, *M. faizahae* n. sp. showed the highest similarity to the *Myxobolus* sp. in this study, particularly in both widths of larger and smaller polar capsules.

### *Thelohanellus gonionoti* n. sp.

**Fig. 4:**
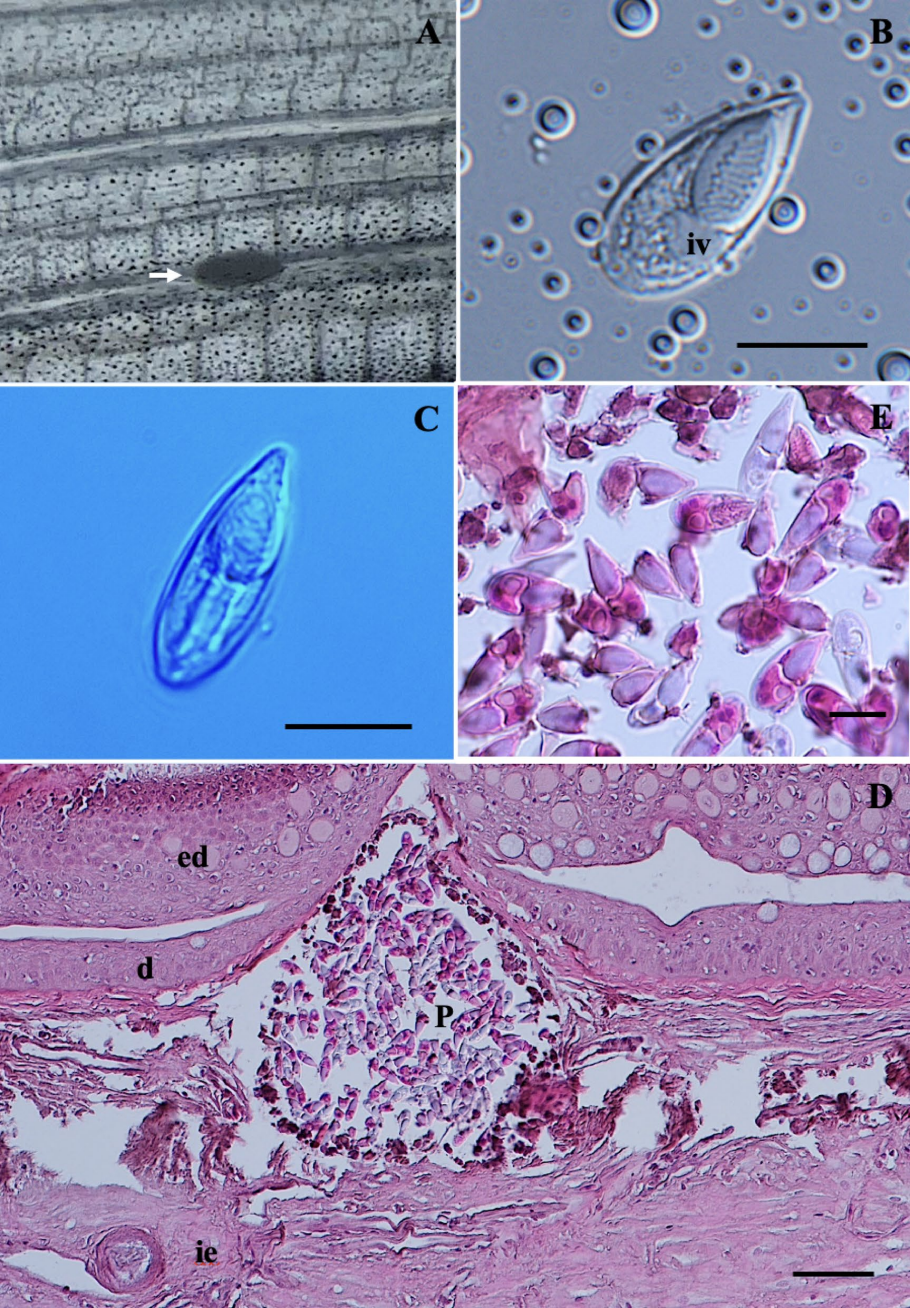
Photomicrographs of *Thelohanellus gonionoti* n. sp. from the fin of *Barbonymus gonionotus*. (**A**) Plasmodium (white arrow) of *T. gonionoti* under the dermis partially covering the fin ray and the interlepidotrichial ligament. (**B**) Spore in frontal view with the presence of an iodinophilous vacuole (iv) within the sporoplasm. (**C**) Spore in sutural view. (**D**) Histological transverse section of the tail fin showing a plasmodium (P) located between the interlepidotrichial ligament (ie) and the dermis (d), and bulging toward the epidermis (ed). (**E**) Higher magnification of the plasmodium (P) filled with mature spores. Stained with hematoxylin and eosin (H&E). Scale bars represent 10 µm, except (**D**) 50 µm, and (**E**) 20 µm.

*Plasmodia*: Ellipsoidal shape plasmodium found under the dermis covering the interlepidotrichial ligament and the fin rays Fig. 4A).

*Description of myxospores*: Fixed spores elongate-pyriform, with tapered and truncated anterior ends in both frontal and sutural views (Fig. 4B–C, 9C) measuring 18.4 ± 1.0 (16.3–18.3) µm in length, 8.9 ± 0.8 (7.2–9.2) µm in width, and 6.9 ± 0.4 (6.4–6.9) µm in thickness (*n* = 5). A single pyriform polar capsule measuring 9.2 ± 1.0 (7.3–10.3) µm in length and 4.8 ± 0.5 (3.8–5.6) µm in width and occupied more than half of the spore body cavity. Polar tubule coiling eight to nine times, positioned perpendicularly to the longitudinal axis of the polar capsules. Sutural line straight, smooth and thick in the middle of spore body. Sporoplasm binucleate measuring 1.1 ± 0.2 (0.7–0.9) in diameter and containing an iodinophilous vacuole measuring 3.8 ± 0.5 (2.8–3.9) in diameter. No mucous envelope observable. Measurements from 30 formalin-fixed spores from one host.

### Taxonomic summary

Type host: Java barb, *Barbonymus gonionotus*.

Locality: Sungai Tong, Setiu, Terengganu, Malaysia.

Site of infection: Under the dermis and above the interlepidotrichial ligament.

Prevalence: 50.0% (3/6).

Type material: Phototypes were deposited in the parasitological collection of the Zoological Department, Hungarian Natural History Museum, Budapest, Coll. No. HNMPCC-HNHM-PAR-72093.

Etymology: The name *Thelohanellus gonionoti* n. sp. was derived from the name of the fish host, *Barbonymus gonionotus*.

Histology: Histopathological analysis of the tail fin revealed a plasmodium with mature spores (Fig. 4E), which located under the dermis and above the interlepidotrichial ligament. The plasmodium was bulging toward the epidermis (Fig. 4D). The dermis and interlepidotrichial tissue are also visible in the histological section.

18S rDNA sequence: Partial 18S rDNA sequence of *T. gonionot*i n. sp., consisting of 1,897 base pairs was deposited in GenBank under the accession number PV665940. The 18S rDNA sequence from the *T. gonionoti* n. sp. did not significantly match any of the myxozoan sequences available in GenBank. Pairwise distance estimation of the newly obtained 18S rDNA sequence indicated the highest similarities by 98.9% to *T. barbonymi* n. sp. (PV665941) (Table 6). Phylogenetic analysis revealed that *T. gonionoti* n. sp. was clustered within a monophyletic clade together with other gill-infecting *Thelohanellus* spp., that parasitize cyprinids such as *B. altus*, *B. gonionotus*, and *Mystacoleucus marginatus* Valenciennes, 1842, with maximum bootstrap support (Fig. 10).

Remarks: The morphology and morphometrics of *T. gonionoti* n. sp. were distinct from those of any previously described *Thelohanellus* spp., characterized by truncated anterior ends (Table 4). The closest morphological resemblance to *T. gonionoti* n. sp. was found to *T. zahrahae* (both previously described and present study) and the newly described* T. barbonymi* n. sp., although notable morphometric differences were present. *Thelohanellus gonionoti* n. sp. can be distinguished from both species by the polar capsule position, which leans towards the side of the spore body. In terms of morphometric measurements, *T. gonionoti* n. sp. differed significantly from the previously described *Thelohanellus* spp. It shared similarities only with *T. barbonymi* n. sp. in terms of the length of the polar capsule and the number of coils.

**Table 4:** Comparative data for myxospore measurements (mean value and standard deviation (SD) followed by the range in parentheses) of *Thelohanellus gonionoti* n. sp., *Thelohanellus barbonymi* n. sp., *Thelohanellus zahrahae* and species with truncate anterior ends. All measurements are in μm. SL spore length, SW spore width, ST spore thickness, PCL polar capsule length, PCW polar capsule width, PFC polar filament coils, D diameter.

Species	Host	Site infection	SL	SW	ST	PCL	PCW	PFC	Reference
***Thelohanellus gonionoti n. sp.***	*Barbonymus gonionotus*	**Fins**	18.4 ± 1.0 (16.3–18.3)	8.9 ± 0.8 (7.2–9.2)	6.9 ± 0.4 (6.4–6.9)	9.2 ± 1.0 (7.3–10.3)	4.8 ± 0.5 (3.8–5.6)	8–9	**Present study**
***Thelohanellus barbonymi n. sp.***	*Barbonymus altus*	**Gill arches**	22.0 ± 0.7 (20.3–23.2)	9.9 ± 0.6 (8.1–10.8)	7.7 ± 0.5 (6.7–8.3)	9.1 ± 0.5 (8.2–10.1)	5.4 ± 0.3 (4.9–5.9)	8	**Present study**
***Thelohanellus zahrahae***	*Barbonymus gonionotus*	**Gill filaments**	20.5 ± 0.5 (19.4–21.6)	9.1 ± 0.6 (8.1–10.6)	7.6 ± 0.2 (7.3–7.8)	8.8 ± 0.8 (6.6–10.2)	5.5 ± 0.5 (4.7–6.8)	7	**Present study**
*T. zahrahae*	*Barbonymus gonionotus*	Gill filaments	23.8 ± 1.3 (21.7–26.3)	9.0 ± 0.3 (8.5–9.4)	7.6 ± 0.1 (7.5–7.9)	9.9 ± 1.0 (7.9–10.8)	6.3 ± 0.5 (5.3–6.6)	7	^24^
*T. catlae*	*Barbonymus gonionotus*	Gill, skin	19.8	9.9	8.2	D: 9.9	–	–	^25^
*T. boggoti*	*Labeo boggut*	Gill lamellae	11.5 (11.0–12.0)	6.8 (6.0–7.5)	–	6.2 (5.5–7.0)	3.8 (3.6–4.0)	10–11	^60^
*T. assambai*	*Labeo* sp.	Gills and fins	10.8 (9.0–12.0)	5.5 (5.0–7.0)	–	6.3 (5.0–7.6)	3.4 (3.0–4.0)	5–6	^5^
*T. lamelliformis*	*Catla catla*	Gill lamellae	10.27	4.9	0.5	3.8	2.6	6–7	^61^

### *Thelohanellus barbonymi* n. sp.

**Fig. 5:**
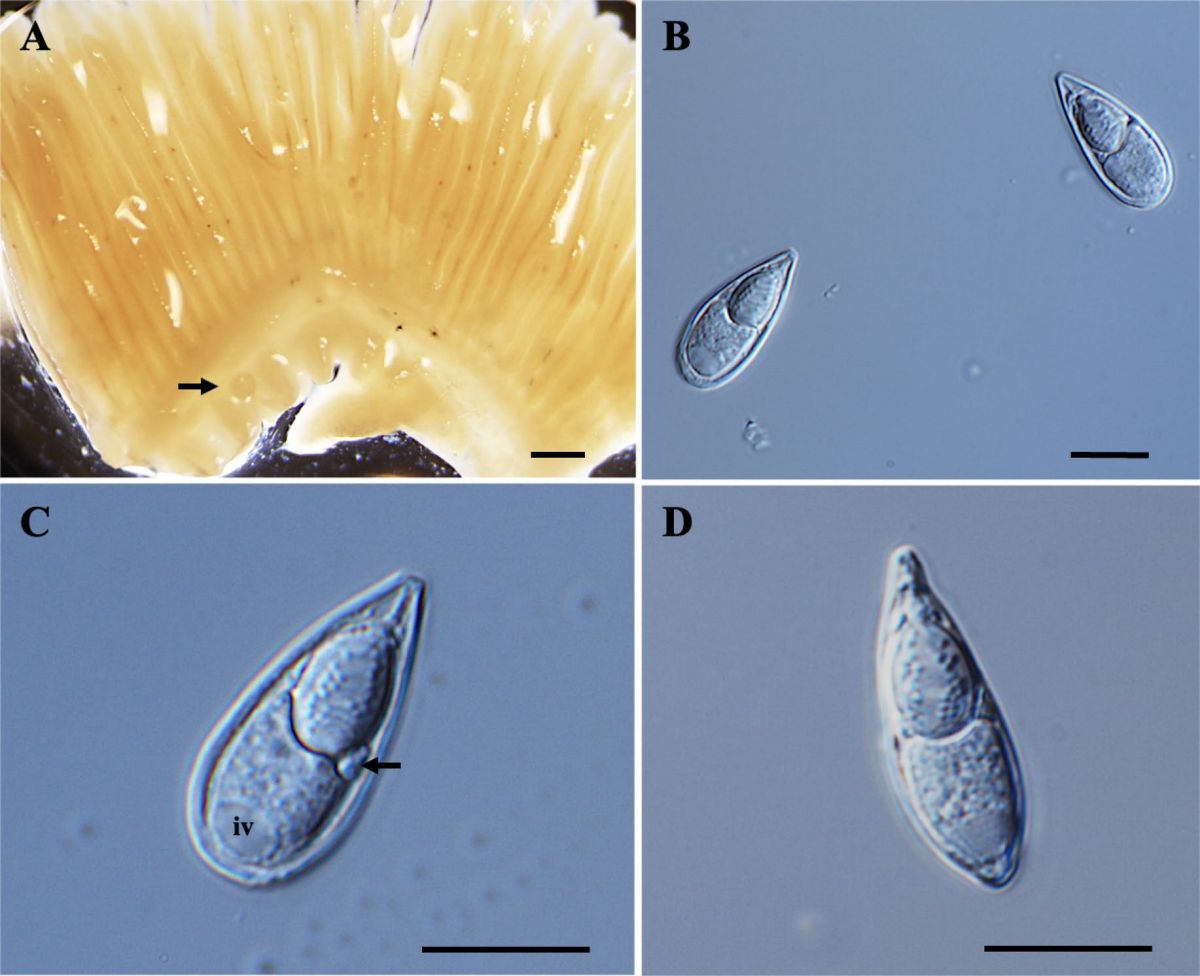
Photomicrographs of *Thelohanellus barbonymi* n. sp. from the gill of *Barbonymus altus*. (**A**) Plasmodium (black arrow) of *T. barbonymi* n. sp. in formalin-fixed gill arches. (**B**) Mature spores of *T. barbonymi* n. sp. released from plasmodium. (**C**) Spore in frontal view with the presence of single nucleus (black arrow) and an iodinophilous vacuole (iv) within the sporoplasm. (**D**) Spore in sutural view. Scale bars represent 10 µm, except (**A**) 500 µm.

*Plasmodia*: Found in the gill arches, histozoic, round to oval in shape (Fig. 5A), measuring 0.22 mm in diameter (*n* = 1).

*Description of myxospores:* Fixed spores elongate-pyriform, with tapered and truncated anterior ends in both frontal and sutural views (Figs. 5B–D, 9D) measuring 22.0 ± 0.7 (20.3–23.2) µm in length, 9.9 ± 0.6 (8.1–10.8) µm in width, and 7.7 ± 0.5 (6.7–8.3) µm in thickness (*n* = 15). A single pyriform polar capsule measuring 9.1 ± 0.5 (8.2–10.1) µm in length and 5.4 ± 0.3 (4.9–5.9) µm in width, occupying ¼ of the spore body cavity. Polar tubule coiling eight times, positioned perpendicularly to the longitudinal axis of the polar capsules. Sutural line straight, smooth and thin in the middle of spore body. Sporoplasm binucleate and containing an iodinophilous vacuole measuring 4.4 ± 0.4 (3.4–4.9) µm in diameter. No mucous envelope observable. Measurements from 30 formalin-fixed spores from one host.

### Taxonomic summary

Type host: Red tailed tinfoil barb, *Barbonymus altus*.

Locality: Sungai Tong, Setiu and Sungai Nerus, Kuala Nerus, Terengganu, Malaysia.

Site of infection: Gill arches.

Prevalence: 12.5% (2/16).

Type material: Phototypes were deposited in the parasitological collection of the Zoological Department, Hungarian Natural History Museum, Budapest, Coll. No. HNMPCC-HNHM-PAR-72092.

Etymology: The name *Thelohanellus barbonymi* n. sp. was derived from the genus name of the fish host, *Barbonymus altus*.

18S rDNA sequence: Partial 18S rDNA sequence of *T. barbonymi* n. sp., consisting of 1,907 base pairs was deposited in GenBank under the accession number PV665941. The 18S rDNA sequence of *T. barbonymi* n. sp. did not significantly match any other myxozoan sequences available in GenBank. Pairwise distance estimation of the newly obtained 18S rDNA sequence indicated the highest similarities by 98.9% to *T. gonionoti* n. sp. (PV665940) (Table 6). Phylogenetic analysis revealed that *T. barbonymi* n. sp. was positioned in a monophyletic clade together with other gill-infecting *Thelohanellus* spp., along with *T. gonionoti* n. sp. that parasitize cyprinids such as *B. altus*, *B. gonionotus*, and *M. marginatus*, with maximum bootstrap support (Fig. 10).

Remarks: The morphology and morphometrics of *T. barbonymi* n. sp. were distinct from any previously described *Thelohanellus* spp., characterized by truncated anterior ends (Table 4). The closest morphological and morphometric resemblance to *T. barbonymi* n. sp. was found with *T. zahrahae*, both in previous and present study, although notable morphometric differences were present. Myxospore of *T. barbonymi* n. sp. was smaller than *T. zahrahae* from previous study by Székely et al.^24^ but larger than *T. zahrahae* from the present study. However, *T. barbonymi* n. sp. was wider than both *T. zahrahae* from previous study and the present study*.* They can be distinguished by having a different number of coils (8 *vs.* 7). Regarding the spore width, *T. barbonymi* n. sp. showed the highest similarities to *T. catlae*, Chakravarty et Basu, 1948. When comparing the newly described *T. gonionoti* n. sp. and *T. zahrahae* from the present study, *T. barbonymi* n. sp. was the largest in size among these species.

***Thelohanellus zahrahae*** Székely et Molnár, 2009

**Fig. 6:**
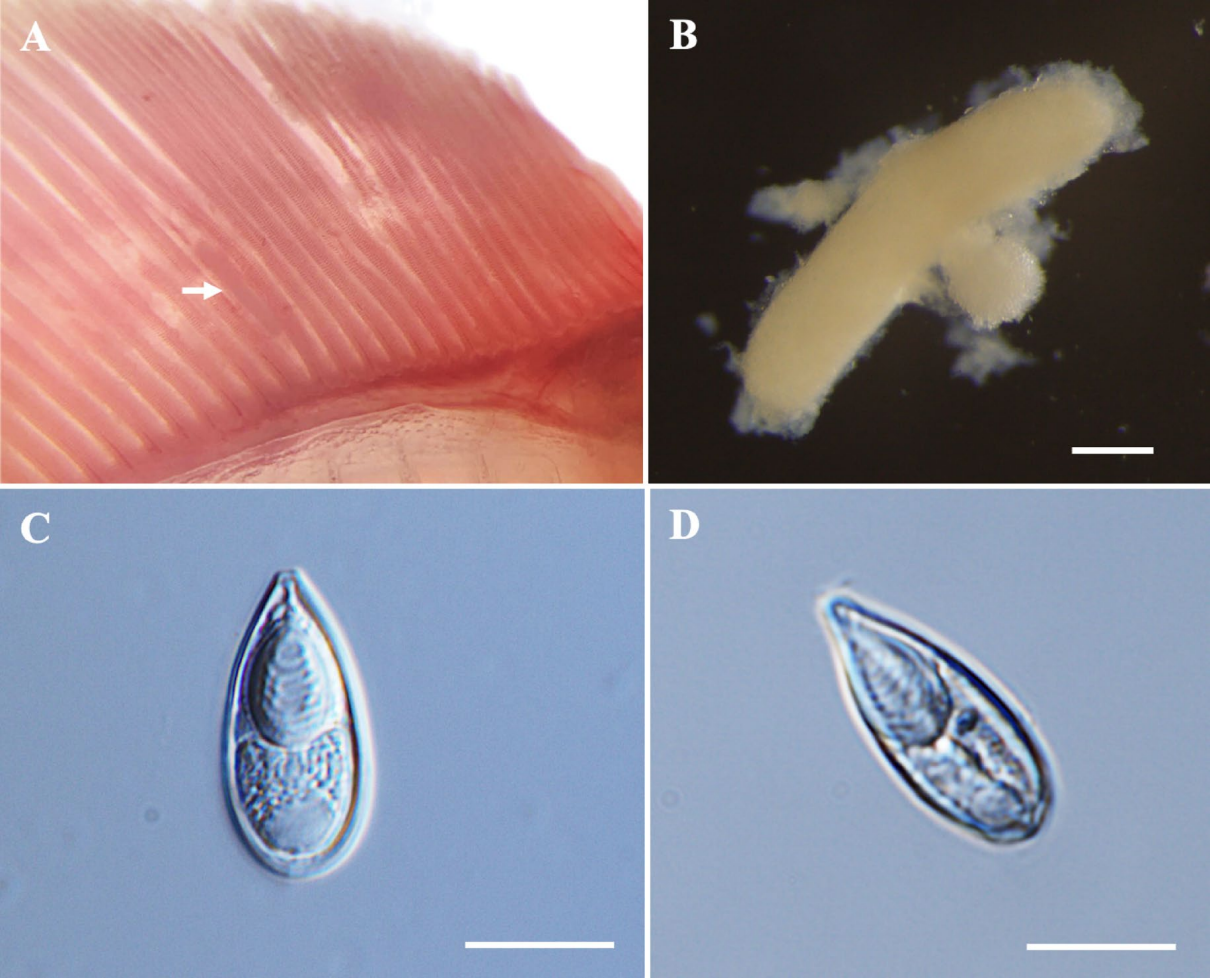
Photomicrographs of *Thelohanellus zahrahae* from the gill of *Barbonymus gonionotus*. (**A**) Large elongated shape plasmodium (white arrow) located inside a filament. (**B**) Higher magnification of the isolated plasmodium of *T. zahrahae*. (**C**) Spore in frontal view. (**D**) Spore in sutural view. Scale bars represent 10 µm, except (**B**) 200 µm.

*Plasmodia*: Found in the gill filaments, histozoic, elongated in shape (Fig. 6A–B), measuring 1.27 ± 7.2 (1.26–1.28) mm in length and 0.29 ± 7.8 (0.28–0.29) mm (*n* = 2).

*Redescription of myxospores*: Fixed spores elongate-pyriform, with tapered and truncated anterior ends in both frontal and sutural views (Fig. 6C–D) measuring 20.5 ± 0.5 (19.4–21.6) µm in length, 9.1 ± 0.6 (8.1–10.6) µm in width, and 7.6 ± 0.2 (7.3–7.8) µm in thickness (*n* = 7). A single pyriform polar capsule measuring 8.8 ± 0.8 (6.6–10.2) µm in length and 5.5 ± 0.5 (4.7–6.8) µm in width, occupied half of the spore body cavity. Polar tubule coiling seven times, positioned perpendicularly to the longitudinal axis of the polar capsules. Sutural line straight, smooth and thick in the middle of spore body. Sporoplasm binucleate measuring 1.0 ± 0.3 (0.6–1.7) in diameter (*n* = 12) and contains an iodinophilous vacuole measuring 3.9 ± 0.5 (3.1–4.8) in diameter. No mucous envelope observable. Measurements from 30 formalin-fixed spores from one host.

### Taxonomic summary

Type host: Java barb, *Barbonymus gonionotus*.

Locality: Sungai Tong, Setiu, Terengganu, Malaysia.

Site of infection: Gill filaments.

Prevalence: 33.3% (2/6).

Type material: Series of phototypes were deposited in the collection of the Fish Pathology and Parasitology Research Team, Veterinary Medical Research Institute, Budapest, Hungary.

18S rDNA sequence: Partial 18S rDNA sequence of *T. zahrahae*, consisting of 1,901 base pairs was deposited in GenBank under the accession number PV647345. The 18S rDNA sequence from the present *T. zahrahae* matched with the sequence of *T. zahrahae* (EU643622) available in GenBank showing 99.7% (Table 6). Phylogenetic analysis revealed that *T. zahrahae* sequences showed a close relationship with an undescribed *Thelohanellus* species (MK332024) infecting *M. marginatus*, with high bootstrap support (Fig. 10).

Remarks: The morphology and morphometric data of *T. zahrahae* were consistent with previously described *T. zahrahae* from Java barb in a fish farm at Machang, Kelantan (Table 4). Although minor differences in size were observed in all measurements, myxospore of *T. zahrahae* from the previous study were generally larger than those from the present study*.* Notably, *T. catlae* was reported by Ky and Te^25^ in Chinh et al.^46^ from the gills and skin of *B. gonionotus*, a common parasite of the common carp (*Cyprinus carpio*). However, significant morphological and morphometric differences were observed, with *T. catlae* is larger in size and possesses pear shaped and spherical polar capsules, in contrast to the present *T. zahrahae,* which has elongated-pyriform spores with truncated anterior ends and a pyriform polar capsule.

***Myxobolus dykovae*** Székely et Molnár, 2009

**Fig. 7:**
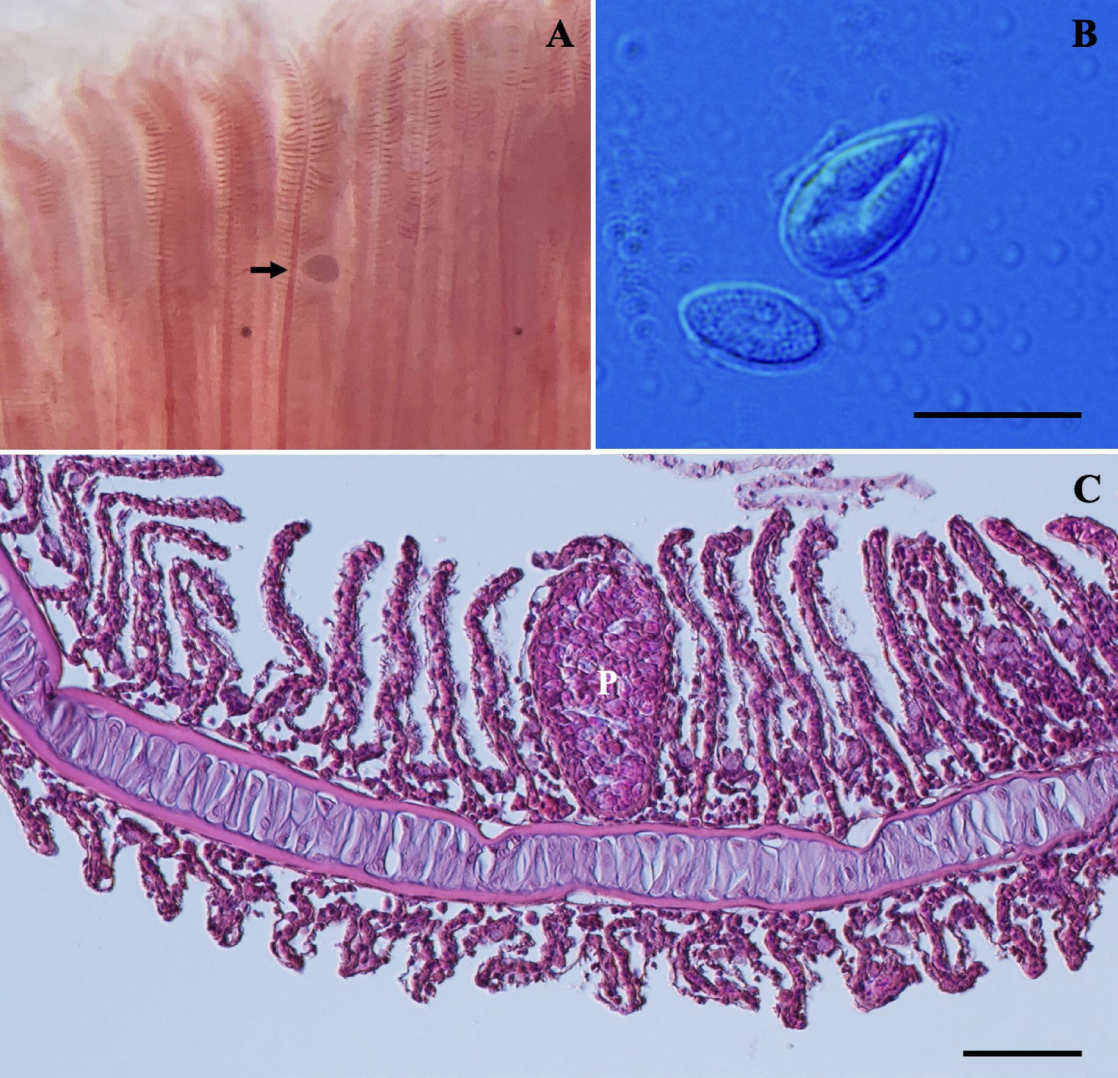
Photomicrographs of *Myxobolus dykovae* from the gill of *Barbonymus schwanefeldii*. (**A**) Plasmodium (black arrow) located in the gills. (**B**) Spore in frontal view. (**C**) Histological transverse section of filament showing plasmodium (P) located between gill lamella of *B. schwanefeldii*, stained with hematoxylin and eosin (H&E). Scale bars represent 10 µm, except (**C**) 50 µm.

*Plasmodia*: Found in the gill lamellae, histozoic, small and oval in shape (Fig. 7A), measuring 0.1 (0.1–0.2) mm in both length and width (*n* = 10).

*Redescription of myxospores*: Fixed spores oval in both frontal and sutural views, tapering at the anterior ends (Fig. 7B) measuring 11.7 ± 0.6 (10.3–12.8) µm in length, 6.8 ± 0.4 (5.9–7.7) µm in width, and 5.4 ± 0.3 (5.1–5.7) µm in thickness (*n* = 4). Two equal, pyriform polar capsules, measuring 5.8 ± 0.5 (4.7–7.0) µm in length and 2.2 ± 0.2 (1.8–2.6) µm in width, and occupied half of the spore body cavity. No intercapsular appendix observed. Polar tubule coil six to seven times and positioned perpendicularly to the longitudinal axis of the polar capsules. Sporoplasm binucleate, containing an iodinophilous vacuole, but no mucous envelope observable. Measurements from 30 formalin-fixed spores from one host.

### Taxonomic summary

Type host: Tinfoil barb, *Barbonymus schwanefeldii*.

Locality: Sungai Tong, Setiu, Terengganu, Malaysia.

Site of infection: Secondary gill lamellae.

Prevalence: 16.6% (1/6).

Type material: Series of phototypes were deposited in the collection of the Fish Pathology and Parasitology Research Team, Veterinary Medical Research Institute, Budapest, Hungary.

Histology: Histopathological analysis showed that an oval-shaped plasmodia filled with mature spores were found intralamellarly within the gills (Fig. 7C).

18S rDNA sequence: Partial 18S rDNA sequence of *M. dykovae*, consisting of 1,643 base pairs was deposited in GenBank under the accession number PV647344. The 18S rDNA sequence from the present *M. dykovae* matched with the sequence of *M. dykovae* available in GenBank. Pairwise distance estimation of the newly obtained 18S rDNA sequence indicated the highest similarities by 99.4% to *M. dykovae* (EU643627) (Table 6). According to the phylogenetic analysis, *M. dykovae* sequences were positioned in a clade comprising many gill-infecting *Myxobolus* spp. and various *Myxobolus* spp. that infect others organs besides gills (Fig. 10).

Remarks: The morphology and morphometric data of *M. dykovae* were consistent with previously described *M. dykovae* from tinfoil barb in Tasik Kenyir (Table 2). Minor differences in size were observed in all characters, but these remained within the established range of variation. Notably, the morphometrics of the present *M. dykovae* showed the highest similarity to *Myxobolus alvarezae* Cech, Molnár et Székely, 2012 in all measurements except spore thickness and polar capsule length.

### *Myxidium* sp.

**Fig. 8:**
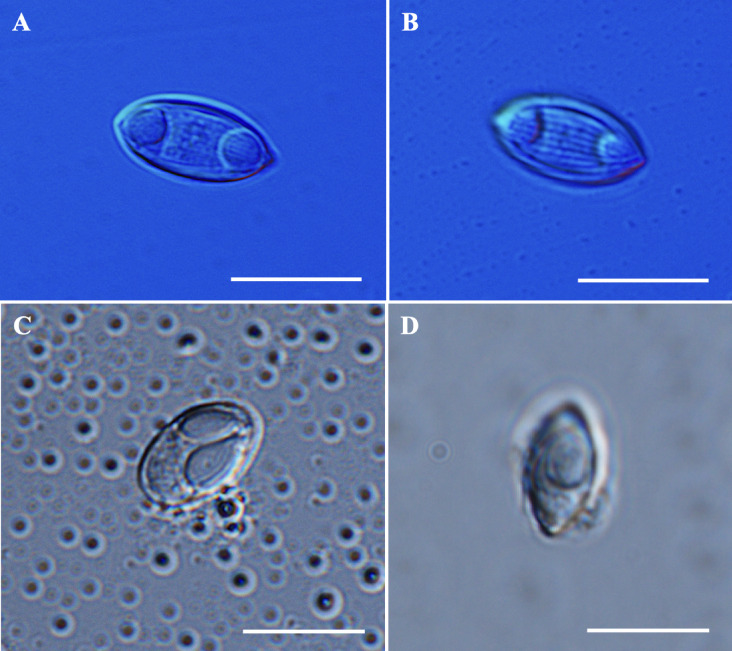
Photomicrographs of *Myxidium* sp. from the gallbladder of *Barbonymus gonionotus and Myxobolus* sp. from the muscle cells of* B. schwanefeldii*. (**A**) Spore of *Myxidium* sp. in frontal view. (**B**) Spore of *Myxidium* sp. in sutural view. (**C**) Spore of *Myxobolus* sp. in frontal view. (**D**) Spore of *Myxobolus* sp. in sutural view. Scale bars represent 10 µm.

*Description of myxospores*. Fixed dispersal spores, fusiform shaped in both frontal and sutural views with tapered ends (Fig. 8A–B), measuring 12.1 ± 0.6 (11.0–13.7) µm in length, 5.9 ± 0.3 (5.2–6.4) µm in width, and 4.8 ± 0.4 (4.2–5.2) µm in thickness (*n* = 4). Two equal, slightly pyriform polar capsules, measuring 3.4 ± 0.2 (3.0–3.7) µm in length and 2.9 ± 0.2 (2.5–3.5) µm in width. Distance between two polar capsules, measuring 4.5 ± 0.6 (3.4–6.0) µm in distance. Polar tubules coiling five times and positioned perpendicular to the longitudinal axis of the polar capsules. Straight sutural line, and the valves exhibiting seven longitudinal striations. Sporoplasm binucleate, filling the spore body cavity between the two polar capsules. Measurements from 30 formalin-fixed spores from two host.

### Taxonomic summary

Type host: Java barb, *Barbonymus gonionotus*.

Locality: Sungai Tong, Setiu, Terengganu, Malaysia.

Site of infection: Gallbladder.

Prevalence: 33.3% (2/6).

Type material: Series of phototypes were deposited in the collection of the Fish Pathology and Parasitology Research Team, Veterinary Medical Research Institute, Budapest, Hungary.

Remarks: The morphology and morphometrics of *Myxidium* sp. did not match any previously described *Myxidium* spp. (Table 5). The closest morphological resemblance to *Myxidium* sp. was found with *Myxidium macropodus* Chen et Hsieh, 1984 and* Myxidium aristichthysi* Chen in Chen and Ma^62^ although notable morphometric differences were present. In terms of morphometric measurements, *Myxidium* sp. showed the greatest similarities to *Myxidium chiluense* Ma in Chen and Ma^62^ in most measurements. Notably, the spore thickness of *Myxidium* sp. was similar to that of *Myxidium mapienense* Ma et Zhao in Chen and Ma^62^. Throughout this study, several attempts at molecular analyses were performed; however, these attempts were unsuccessful due to the low number of spores available.

**Table 5:** Comparative data for myxospore measurements (mean value and standard deviation (SD) followed by the range in parentheses) of *Myxidium* sp. and species with fusiform to ellipsoidal shaped. All measurements are in μm. SL spore length, SW spore width, ST spore thickness, PCL polar capsule length, PCW polar capsule width, PFC polar filament coils, SV valvular striations.

Species	Host	Site infection	SL	SW	ST	PCL	PCW	PFC	SV	Reference
***Myxidium* sp.**	***Barbonymus gonionotus***	**Gallbladder**	12.1 ± 0.6 (11.0–13.7)	5.9 ± 0.3 (5.2–6.4)	4.8 ± 0.4 (4.2–5.2)	3.4 ± 0.2 (3.0–3.7)	2.9 ± 0.2 (2.5–3.5)	5	7	**Present study**
*M. chiluense*	*Abbotina rivularis, Anabarilius grabami*	Gallbladder	12.4 (12.0–13.6)	6.2 (5.8 – 6.6)	5.9 (5.8–6.2)	3.2 (3.0–3.2)	2.8 (2.6–3.0)	–	Several	^62^
*M. mapienense*	*Leiobagrus marginatus*	Kidney	12.6 (12.0–12.8)	4.9 (4.8–5.2)	4.9	3.7 (3.2–4.0)	2.2 (1.6–2.4)	–	–	^62^
*M. tongrenense*	*Gnathopogon argentatus*	Gallbladder	11.0 (10.5–12.0)	6.0 (5.5–6.5)	–	3.6 (3.4–4.0)	3.7 (3.2–4.0)	–	8–10	^62^
*M. macropodus*	*Macropodus chinensis*	Gallbladder	13.2 (12.0–14.0)	6.8 (6.2–7.2)	6.0 (6.0–6.2)	3.7 (3.6–3.8)	3.6 (3.4–3.6)	–	7–8	^4^
*M. aristichthysi*	*Aristichthys nobilis*	Gallbladder	13.9 (12.1–14.4)	6.0 (5.8–6.2)	–	4.9 ± 0.8 (3.7–5.2)	4.2 ± 0.5 (3.0–4.5)	–	8–11	^4^

### *Myxobolus* sp.

*Plasmodia*: Found in the muscle cells, histozoic, elongated in shape.

*Description of myxospores*: Fixed spores oval in frontal view and lemon-shaped in sutural view (Fig. 8C–D), measuring 11.7 ± 0.5 (11.0–12.5) µm in length, 6.4 ± 0.5 (5.8–7.2) µm in width, and 4.8 ± 0.8 (4.3–5.4) µm in thickness (*n* = 2). Two pyriform and unequal polar capsules; larger polar capsule, measuring 6.0 ± 0.5 (5.3–6.9) µm in length and 2.8 ± 0.3 (2.2–3.2) µm in width and smaller polar capsule measuring 4.8 ± 0.4 (4.2–5.4) µm in length and 2.2 ± 0.3 (1.7–2.9) µm in width. No intercapsular appendix observed. Polar tubule coil five times in larger polar capsules and four times in smaller polar capsule, positioned perpendicularly to the longitudinal axis of the polar capsules. Sporoplasm binucleated, but iodinophilous vacuole not visible and no mucous envelope observable. Measurements from 12 formalin-fixed spores from one host.

**Fig. 9:**
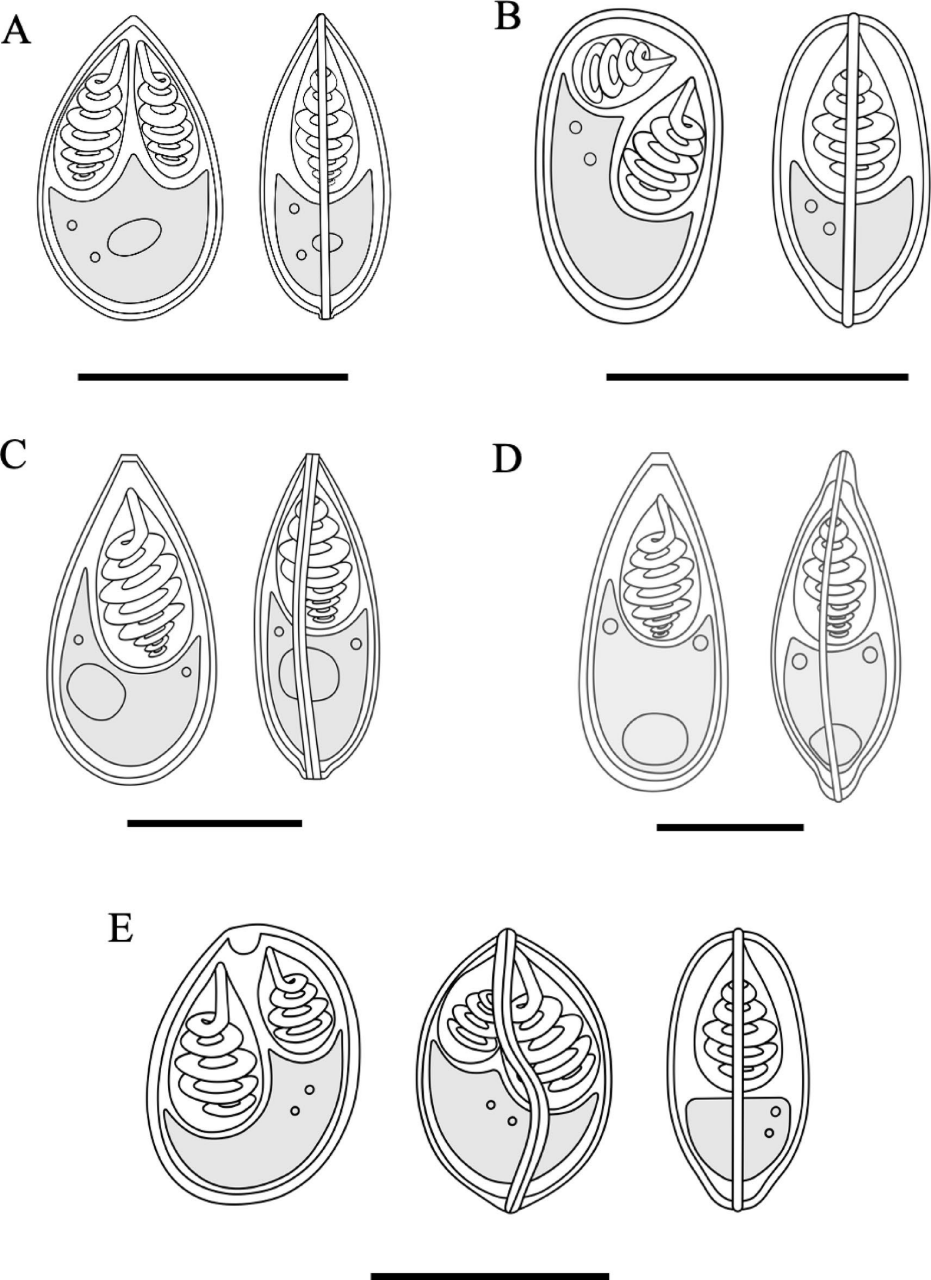
Schematic drawings of myxosporean parasites from *Barbonymus* species. (**A**) *Myxobolus gonionoti* n. sp. in frontal and sutural views. (**B**) *Myxobolus faizahae* n. sp. in frontal and sutural views. (**C**) *Thelohanellus gonionoti* n. sp. in frontal and sutural views. (**D**) *Thelohanellus barbonymi* n. sp. in frontal and sutural views. (**E**) *Myxobolus barbonymi* n. sp. in frontal, sublateral and sutural views. Scale bars represent 10 µm.

### Taxonomic summary

Type host: Tinfoil barb, *Barbonymus schwanefeldii*.

Locality: Sungai Tong, Setiu, Terengganu, Malaysia.

Site of infection: Intracellularly of skeletal muscle cells.

Prevalence: 16.6% (1/6).

Type material: Series of phototypes were deposited in the collection of the Fish Pathology and Parasitology Research Team, Veterinary Medical Research Institute, Budapest, Hungary.

Remarks: The morphology and morphometrics of *Myxobolus* sp. did not match any previously described *Myxobolus* spp. (Table 3). The closest morphological and morphometric resemblance to *Myxobolus* sp. was found with *M. pseudodispar* although notable morphometric differences were present. Notably, the morphometrics of *Myxobolus* sp. showed the greatest similarities to *M. musculi* Keysselitz, 1908 in terms of spore length, and to *M. bhadrensis* in all measurements except for the width of the larger polar capsule. Unfortunately, molecular analysis of this sample was unsuccessful due to low number of spores available.

## Discussion

This study identified five novel myxozoan species (three *Myxobolus* spp., two *Thelohanellus* spp.), detected two previously described species (*M. dykovae*, and *T. zahrahae*) and recorded two unidentified species (*Myxobolus* sp. and *Myxidium* sp.) from *Barbonymus* spp., based on analyses of the 18S rDNA gene sequences and morphological characteristics. The species’ tissue preferences were different, with three *Myxobolus* spp. infecting the muscle cells, a *Myxobolus* and two *Thelohanellus* species—the gills, a *Thelohanellus* species—the fins and a *Myxidium* sp.—the gallbladder.

Due to the high similarity of *Myxobolus* spp. spores, species identification based solely on morphological criteria is challenging. While host specificity and the predilection site of plasmodia within organs or tissues can assist in distinguishing morphologically identical or similar spores, accurate differentiation often requires molecular analyses. This approach is particularly critical in cases where the host species are closely related, such *B. gonionotus*, *B. schwanefeldii,* and *B. altus*, which fishes may be susceptibility to the same myxosporean species.

**Fig. 10:**
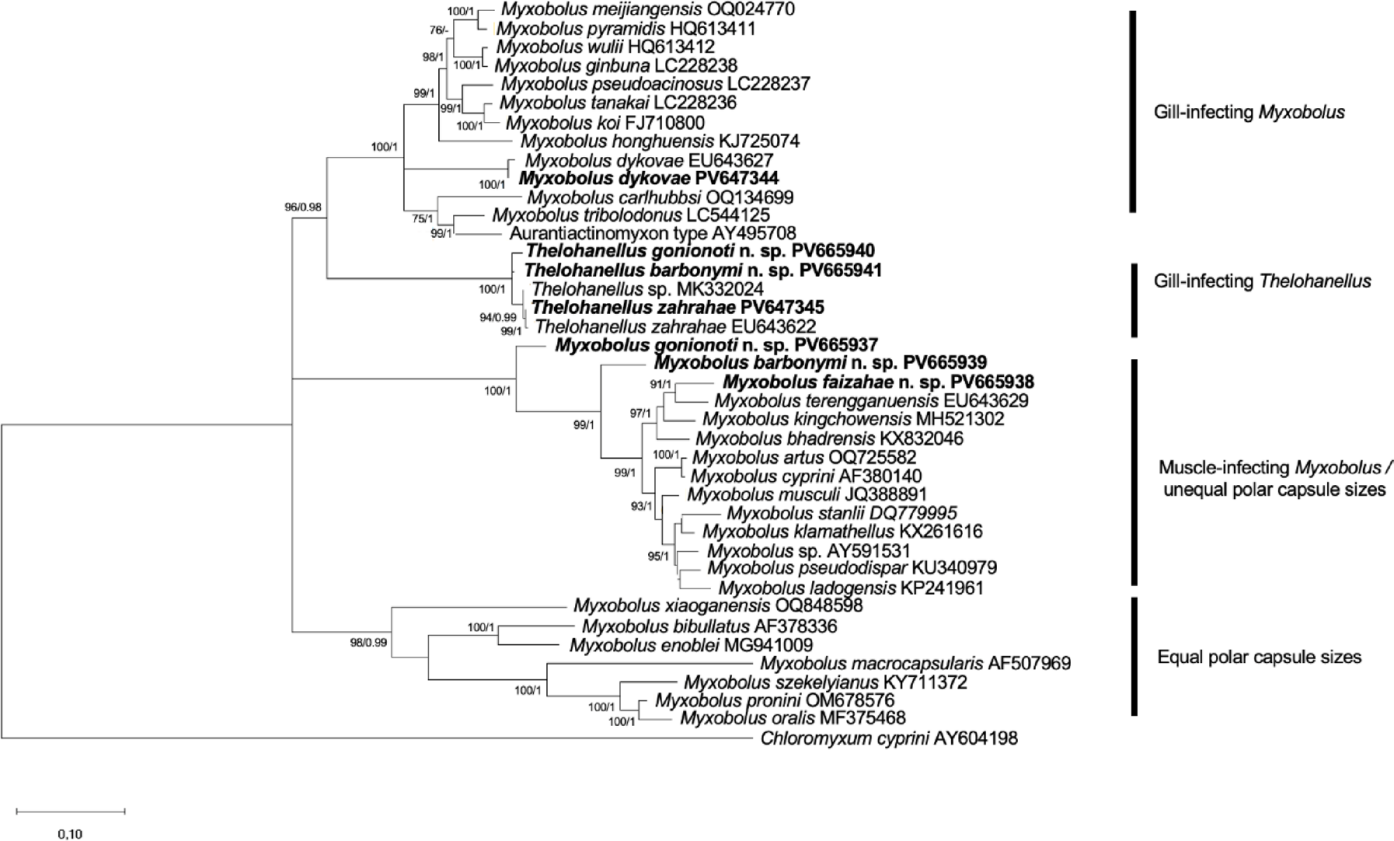
Maximum likelihood phylogenetic tree of 18S rDNA sequences of *Myxobolus gonionoti* n. sp., *Myxobolus barbonymi* n. sp., *Myxobolus faizahae* n. sp., *Thelohanellus gonionoti* n. sp., *Thelohanellus barbonymi* n. sp., *Myxobolus dykovae*, *Thelohanellus zahrahae,* and related species. *Chloromyxum cyprini* was chosen as outgroup. Nodal supports are indicated for maximum likelihood (ML) at 1000 replicates and Bayesian Inference (BI). Only values with ≥ 70% bootstrap (BS) and ≥ 0.90 posterior probabilities (PP) support are presented. The sequences from the present study is in bold. The scale bar indicates the expected number of substitutions per site.

Organ and tissue specificity is also a crucial diagnostic criterion for myxosporean species^55^. Besides gills, muscle cells are also a common infection site for various myxosporean parasites in fish^63^. As of 2021, approximately 75 of 979 *Myxobolus* spp. have been described from muscle tissue^3,58,64^. The majority of these muscle-dwelling species, such as *M. cyprini, M. musculi* and *M. pseudodispar*, belong to the ‘pseudodispar’ morphological type and form intracellular plasmodia. The *Myxobolus* spp. (*M. barbonymi* n. sp., *M. faizahae* n. sp. and *Myxobolus* sp.) found in this study also exhibit ‘pseudodispar’-like morphology. Notably, one of the three species, *M. faizahae*, formed intramuscular plasmodia, whereas *M. barbonymi* n. sp. and *Myxobolus* sp. developed intracellular plasmodia, resembling those of *M. pseudodispar*. Phylogenetic analysis and pairwise distance calculations confirmed the validity of *M. barbonymi* n. sp. and *M. faizahae* n. sp. as new species. Our results indicate that the phylogenetic relationships among *Myxobolus* spp. in this study show some correlation with their predilection sites in the host. Specifically, the newly identified *Myxobolus* spp. that infect muscle tend to cluster together in one clade (Fig. 10). This finding is consistent with previous studies that highlight the importance of tissue tropism as a phylogenetic criterion for myxosporean species^36,65,66^. Notably, the two unequal polar capsules also appear to be relevant for the phylogenetic relationships of *Myxobolus* species in this study. The newly identified *M. barbonymi* n. sp. and *M. faizahae* n. sp., along with other *Myxobolus* species having two unequal polar capsules (except for *Myxobolus stanlii* Iwanowicz, Iwanowicz, Howerth, Schill, Blazer et Johnson, 2013, which has equal polar capsule sizes), were clustered within muscle-infecting *Myxobolus* spp. (Fig. 10). Conversely, those with two equal polar capsules tended to cluster within a smaller group of *Myxobolus* spp. (Fig. 10).

The gills are the most commonly affected organs by myxosporean parasites^67^. This study identified two newly described gill-infecting myxosporeans: *M. gonionoti* n. sp. and *T. barbonymi* n. sp., alongside two previously described species *M. dykovae* and *T. zahrahae. Myxobolus gonionoti* n. sp. was compared with all known gill-infecting *Myxobolus* species from the literature, and exhibits the closest morphological resemblance to *M. dykovae*. Additional comparisons with related species, including *M. sangei* and *M. carlhubbsi,* also revealed distinguishing features that support the designation of *M. gonionoti* n. sp. as a new species. Interestingly, *M. gonionoti* n. sp. may be confused with *M. macrocapsularis*, a species previously reported from *B. gonionotus* by Ky and Te^25^ in Chinh et al.^46^. However, molecular analysis clearly differentiates *M. gonionoti* n. sp. from *M. macrocapsularis*, revealing only 77% sequence similarity (Table 6), which strongly supports its recognition as a distinct species. It is important to note that earlier identifications of *M. macrocapsularis* were based solely on morphological features, as molecular data were not widely used at the time. Therefore, the spores reported by Ky and Te^25^ may have been misidentified, and it is plausible that they were actually observing *M. gonionoti* n. sp.

**Table 6:** Pairwise distances and sequence similarities (%) for the 18S rDNA of *Myxobolus gonionoti* n. sp., *Myxobolus barbonymi* n. sp., *Myxobolus faizahae* n. sp., *Thelohanellus gonionoti* n. sp., *Thelohanellus barbonymi* n. sp., *Myxobolus dykovae*, *Thelohanellus zahrahae,* and closely related species. The sequence from the present study is in bold.

Sequence	Genetic distance	Sequence similarities (%)
*Myxobolus gonionoti* n. sp. PV665937		
*** Myxobolus barbonymi* n. sp. PV665939**	0.088	91.2
* Myxobolus macrocapsularis* AF507969	0.230	77.0
*Myxobolus barbonymi* n. sp. PV665939		
* Myxobolus pseudodispar* KU340979	0.073	92.7
* Myxobolus musculi* JQ388891	0.074	92.6
* Myxobolus klamathellus* KX261616	0.077	92.3
* Myxobolus bhadrensis* KX832046	0.079	92.1
* Myxobolus* sp. AY591531	0.079	92.1
*Myxobolus faizahae* n. sp. PV665938		
* Myxobolus bhadrensis* KX832046	0.056	94.4
* Myxobolus kingchowensis* MH521302	0.058	94.2
* Myxobolus cyprini* AF380140	0.067	93.3
* Myxobolus pseudodispar* KU340979	0.068	93.2
* Myxobolus klamathellus* KX261616	0.069	93.1
* Myxobolus artus.* OQ725582	0.069	93.1
*Theohanellus barbonymi* n. sp. PV665941		
*** Theohanellus gonionoti* n. sp. PV665940**	0.011	98.9
* Thelohanellus* sp. MK332024	0.014	98.6
*** Thelohanellus zahrahae* PV647347**	0.017	98.3
* Thelohanellus zahrahae* EU643622	0.017	98.3
*Thelohanellus zahrahae* PV647345		
* Thelohanellus zahrahae* EU643622	0.003	99.7
* Myxobolus dykovae* PV647344		
* Myxobolus dykovae* EU643627	0.006	99.4

Plasmodia of *T. barbonymi* n. sp. developed in the gill arches, an unusual location for *Thelohanellus* species, which typically develop in gill lamellae and gill filaments. Although *T. barbonymi* n. sp. shares similarities with *T. zahrahae*, which also infects the gills of different host within the same genus, it represents a distinct taxon based on the genetic characteristics and host species. Notably, only a few *Thelohanellus* species, such as *Thelohanellus valeti* Fomena et Bouix, 1987, have been reported from both stomach and gill arches of *Barbus jae* and *Barbus aspilus* from Cameroon, corroborated the unusual site preference observed in *T. barbonymi* n. sp. These findings highlight the diversity of tissue tropism among *Thelohanellus* species and support the acceptance of *T. barbonymi* n. sp. as a novel taxon.

This study also identified previously described gill-infecting myxozoan species including *T. zahrahae* and *M. dykovae*. Both species were identified based on morphological characteristics consistent with earlier descriptions and then further validated by molecular analysis. Although other *Thelohanellus* species, such as *T. catlae*, have previously been reported from *B. gonionotus,* as noted by Ky and Te^25^ in Chinh et al.^46^, our findings indicate that the present *T. zahrahae* differs notably in key features. Additionally, the most significant difference is with the shape of the plasmodia; *T. zahrahae* has elongated plasmodia (Fig. 6A–B), while *T. catlae* has spherical-shaped plasmodia confirming the distinct identity of *T. zahrahae*. Furthermore, molecular analysis of 18S rDNA sequences confirmed the identity of our samples as *T. zahrahae* and *M. dykovae*, supporting their classification despite the slight morphological differences.

The phylogenetic relationships among *Myxobolus* spp. and *Thelohanellus* spp. in this study show some correlation with their predilection sites in the host. It is noteworthy that *M. dykovae* clustered with other predominantly gill-infecting *Myxobolus* spp., as well as some species of *Myxobolus* spp. that infect other organs such as pharynx and hepatopancreas (Fig. 10). However, *M. gonionoti* n. sp., in contrast, is clustered with muscle-infecting *Myxobolus* spp. (Fig. 10). Its unique position can be explained by the absence of closely related species that also infect gills in current genetic databases, suggesting that such species from that geographic area are still undiscovered or undescribed. It is also plausible that these parasites evolved in parallel with muscle parasites but later adapted to infect gills^68^. Regarding the *Thelohanellus* lineage, *T. barbonymi* n. sp. and *T. zahrahae* cluster with other gill-infecting *Thelohanellus* spp. (Fig. 10). *Thelohanellus barbonymi* n. sp. forms a close relationship with *T. gonionoti* n. sp., which infects the fins of *B. barbonymus*. Meanwhile, *T. zahrahae* forms a sister group with *Thelohanellus* sp. (MK332024) from *M. marginatus*. This pattern highlights the complex phylogenetic connections within *Thelohanellus*, which often exhibit polyphyly and intermix with *Myxobolus* clades.

The morphometrics of *T. gonionoti* n. sp. were distinct from those of any previously described *Thelohanellus* spp., including those characterized by truncated anterior ends (Table 4). *Thelohanellus gonionoti* n. sp. possesses a unique feature; the polar capsule position leans towards the side of the spore body, in contrast to *T. barbonymi* n. sp. and *T. zahrahae*. There are relatively several fin-infecting *Thelohanellus* spp., including *Thelohanellus assambai* Fomena, Marqués, Bouix et Njine, 1994; *Thelohanellus avijiti* Basu et Haldar, 2003; *Thelohanellus caudatus* Pagarkar et das, 1993; *Thelohanellus disporomorphus* Basu, Modak et Haldar, 2006; *Thelohanellus globulosa* Singh et Kaur, 2012; *Thelohanellus habibpuri* Acharya et Dutta, 2007; *Thelohanellus kalavatae* Singh et Kaur, 2013; *Thelohanellus leshanensis* Zhao et Ma, 1992; *Thelohanellus nikolskii* Akhmerov, 1955; *Thelohanellus potaili* Lalitha Kumari, 1969; *Thelohanellus sanagaensis* Fomena, Marqués, Bouix et Njine, 1994; *Thelohanellus shaochingensis* Chen in Chen et Ma, 1998; *Thelohanellus shortii* Qadri, 1967; *Thelohanellus wusihensis* Chen in Chen et Ma, 1998; *Thelohanellus deri* Singh et Kaur, 2012; *Thelohanellus haldari* Singh et Kaur, 2012; *Thelohanellus rohi* Singh et Kaur, 2015^5,69^. Among these, *T. assambai* from *Labeo* sp., is the only species that possesses truncated anterior ends. Unfortunately, no nucleotide sequence of *T. assambai* is available for comparison with *T. gonionoti* n. sp. to identify its position on the phylogenetic tree*.* Furthermore, 18S rDNA sequence and pairwise distance analyses have confirmed that *T. gonionoti* n. sp. is a valid new species (Table 6). Phylogenetic analyses have revealed that *T. gonionoti* n. sp. clusters within the gill-infecting *Thelohanellus* spp. clade consisting *T. barbonymi* n. sp., *Thelohanellus* sp. and *T. zahrahae* (Fig. 10). Geographical location can also serve as an important criterion for phylogenetic relationships. Notably, all *Thelohanellus* species in this study, which are collected from Southeast Asia, formed a cohesive cluster. Specifically, *T. gonionoti* n sp., *T. barbonymi* n. sp., *T. zahrahae* were identified in Malaysia, while *Thelohanellus* sp. was found in Thailand.

Approximately 79 freshwater *Myxidium* species have been reported from Asia, including India, Japan and China^4^. However, there are only three known *Myxidium* spp. in Southeast Asia, including *Myxidium cf. notopterum* from the gallbladder of *Notopterus notopterus*^70^, *Myxidium* sp. from the gallbladder of *Osteochilus hasselti*^71^, and *Myxidium cuneiforme* from the gallbladder of *B. gonionotus*^72^. *Myxidium* sp. found in the gallbladder of *B. gonionotus* in this study does not match any previously described *Myxidium* spp. Previously, Thumvittayakul et al.^72^ reported *M. cuneiforme*, a common parasite of the common carp *(Cyprinus carpio)* from the gallbladder of *B. gonionotus* in Thailand. When comparing our spores with *M. cuneiforme*, significant differences were observed in both morphology and morphometric measurements. *Myxidium cuneiforme* is larger and possesses crescent-shaped spores with teardrop-shaped polar capsules and 6 to 8 longitudinal striations. In contrast, the present *Myxidium* sp. has fusiform spores with a slightly pyriform polar capsule and seven longitudinal striations. Although, the morphology and morphometrics of the present *Myxidium* sp. are distinct from all previously described *Myxidium* spp., it cannot be described as a new species due to a lack of molecular data.

In conclusion, this study identified nine myxozoan parasites, including five new myxosporeans in *Barbonymus* species collected from freshwater ecosystems in Malaysia. These findings highlight the underexplored diversity of myxosporeans in *Barbonymus* spp. within Malaysian freshwater ecosystems. This research underscores the need for further research to explore more myxosporean parasites from other fish species in Malaysia, contributing to a more comprehensive understanding of the diversity of Malaysian myxozoan fauna.

## Data Availability

The datasets generated and/or analysed during the current study are available in GenBank database under the accession numbers of PV647344, PV647345, PV665937, PV665938, PV665939, PV665940, and PV665941. Type materials have been deposited in the parasitological collection of the Zoological Department, Hungarian Natural History Museum, Budapest, under collection numbers HNMPCC-HNHM-PAR-72089 to HNMPCC-HNHM-PAR-72093, and are available upon reasonable request through the Head of the Zoological Department.
